# Neurons Underlying Aggression-Like Actions That Are Shared by Both Males and Females in *Drosophila*

**DOI:** 10.1523/JNEUROSCI.0142-24.2024

**Published:** 2024-09-24

**Authors:** Liangyu Tao, Deven Ayambem, Victor J. Barranca, Vikas Bhandawat

**Affiliations:** ^1^School of Biomedical Engineering and Health Sciences, Drexel University, Philadelphia, Pennsylvania 19104; ^2^Swarthmore College, Swarthmore, Pennsylvania 19063

**Keywords:** aggression, behavior, connectomics; *Drosophila*

## Abstract

Aggression involves both sexually monomorphic and dimorphic actions. How the brain implements these two types of actions is poorly understood. We found that in *Drosophila melanogaster*, a set of neurons, which we call CL062, previously shown to mediate male aggression also mediate female aggression. These neurons elicit aggression acutely and without the presence of a target. Although the same set of actions is elicited in males and females, the overall behavior is sexually dimorphic. The CL062 neurons do not express *fruitless*, a gene required for sexual dimorphism in flies, and expressed by most other neurons important for controlling fly aggression. Connectomic analysis in a female electron microscopy dataset suggests that these neurons have limited connections with *fruitless* expressing neurons that have been shown to be important for aggression and signal to different descending neurons. Thus, CL062 is part of a monomorphic circuit for aggression that functions parallel to the known dimorphic circuits.

## Significance Statement

Aggression is an important component of social interaction in most animals. Aggressive behaviors serve a critical purpose by helping an animal secure territory, mates, or food. Aggressive behaviors are very diverse in both their goals and their target. However, most studies aimed at uncovering neural circuits important for aggression have found circuits that are sexually dimorphic and are either only present in either male or female or only produce aggression in one. In this study, using *Drosophila* as a model, we report that a small set of neurons, when activated, produce aggressive behaviors in both males and females. We also show that these neurons are not strongly connected to other aggression-promoting neurons implying that many parallel pathways mediate aggression.

## Introduction

Most animals display sexually dimorphic behaviors (Darwin, 1874). One such behavior is aggression, which is important for defending and obtaining different resources necessary for survival and reproduction (Lorenz, 1963). Circuits underlying aggression have been studied in many organisms, including primates, rodents, and flies (Nelson and Trainor, 2007; Hoopfer, 2016; Lischinsky and Lin, 2020). Perhaps due to the high degree of behavioral dimorphism, much of what we know about the neural circuitry underlying aggression has been studied in a sex-specific manner (Dulac and Kimchi, 2007; Manoli et al., 2013; Yamamoto and Koganezawa, 2013; C. F. Yang and Shah, 2014; Asahina, 2018). Yet, aggression is not fully dimorphic, as both vertebrates (Scott, 1966) and invertebrates (Nilsen et al., 2004) show monomorphic (shared by both sexes) motor actions during aggression (Hashikawa et al., 2018; Pandolfi et al., 2021). How neural circuits are organized to drive these shared aspects of aggression is largely unknown.

Like other animals, *Drosophila* exhibits both sexually dimorphic and shared actions during aggression. Sexually dimorphic actions include male-specific lunging and boxing as well as female headbutting and shoving (Sturtevant, 1915; Chen et al., 2002; Nilsen et al., 2004); sexually shared actions include wing threats, charging, approach, standing still, and fencing actions during aggression. While these actions are shared, there is still sexual dimorphism in the details of how they are deployed (Vrontou et al., 2006). For instance, female wing threats are of shorter duration than those of males (Nilsen et al., 2004). Due to the dimorphism in action implementation despite the commonality in the action itself, it is unknown if these actions are driven by sexually dimorphic neurons and circuits or through a common set of neurons within a potentially dimorphic circuit.

Much of the work dissecting the neural circuits underlying fly aggression has focused on dimorphic neurons with a particular focus on neurons involved in male aggression. Most of these neurons express the sex-determination genes *fruitless*(*fru*) and/or *doublesex*(*dsx*) (Vrontou et al., 2006; Siwicki and Kravitz, 2009; Koganezawa et al., 2016; Wohl et al., 2020). In particular, the P1a cluster of *fru*^+^ and *dsx*^+^ neurons plays a role in promoting both male mating and male aggression (Anderson, 2016). Neurons in this cluster function by inducing a persistent intrinsic state, lasting for minutes (Hoopfer et al., 2015), which in the presence of another male fly leads to persistent aggression. The neurons that carry signals downstream of P1a are themselves *fru*^+^ (Y. Jung et al., 2020). Other neurons, all dimorphic, have been found to enhance actions due to aggression through a variety of neurotransmitters, including tachykinin (Asahina et al., 2014), drosulfakinin (Wu et al., 2020), and octopamine (Watanabe et al., 2017). Recently another set of neurons that are not *fru*^+^ have been found to elicit aggressive behaviors (Duistermars et al., 2018); these neurons appear to be functionally unrelated to the *fru*^+^ population. The *fru*^+^/*dsx*^+^ population that contains the P1a neurons is also important for aggression in females. Females lack P1a neurons but have other neurons within this cluster called pC1; one subset in this cluster called the pC1d (which are *dsx*^+^) elicits aggressive behaviors in females. Through its strong and recurrent connections with aIPg neurons (which are *fru*^+^), pC1d neurons (which were initially described as auditory interneurons) and aIPg together drive female-specific aggressive behavior including headbutting and shoving (Deutsch et al., 2020; Schretter et al., 2020; Chiu et al., 2021, 2023). Thus, much progress has been made in understanding the basis of aggression that is dimorphic. In contrast, relatively little is known about monomorphic aggression circuits in *Drosophila*. A recent study identified a set of sexually conserved neurons (Chiu et al., 2021), but these neurons mediate aggressive approaches rather than consummatory actions themselves and the aggressive actions elicited downstream occur through the same sexually dimorphic circuits described above.

Here, we report a set of monomorphic neurons that, upon optogenetic activation, elicit a common set of aggressive actions in both male and female flies. In this study, we aim to answer three questions—what actions are evoked, which neurons are involved, and how do these neurons connect to other neurons associated with aggression. To answer the first question, we quantified the temporal progression of actions. We show that despite driving a common set of actions, there is sexual dimorphism in the temporal progression of each action. Second, we developed an experimental preparation that allows us to drive some aggressive actions in head-fixed flies through spatially targeted optogenetics. Using this setup, we identify the neurons responsible for driving these behaviors. Third, we utilize an EM dataset to show that different subsets of these neurons connect to different descending neurons (DNs) in a modular manner, which suggests the presence of parallel descending motor pathways in driving actions. These neurons also appear to have only sparse connections with the previously reported dimorphic neurons and therefore likely function independently.

## Materials and Methods

### Contact for reagent and resource sharing

Further information and requests for resources and reagents should be directed to and will be fulfilled by the lead contact, Dr. Vikas Bhandawat (vb468@drexel.edu).

### Data and software availability

Data and analysis code supporting the study will be available after publication (as listed in Table 1). Any additional information required to reanalyze the data is available from the corresponding author upon request.

**Table 1.** Resources including flies, reagents and solftware

Reagent or resource	Source	Identifier
Chemicals, peptides, and recombinant proteins		
All-*trans*-retinal	Sigma-Aldrich (Millipore Sigma)	Catalog #R2500-1G
Chicken anti-GFP	Abcam	Catalog #ab13970; RRID: AB_300798
Mouse anti-NC82	Developmental Studies Hybridoma Bank	Catalog #nc82; RRID: AB_2314866
Alexa Fluor 488 anti-chicken	Thermo Fisher Scientific (Invitrogen)	Catalog #A11039, RRID: AB_2534096
Alexa Fluor 633 anti-mouse	Thermo Fisher Scientific (Invitrogen)	Catalog #A21050, RRID: AB_2535718
Dylight 550 anti-FLAG	Thermo Fisher Scientific (Invitrogen)	Catalog #MA1-91878-D550; RRID: AB_1957945
Dylight 488 anti-HA	Thermo Fisher Scientific (Invitrogen)	Catalog #26183-D488; RRID: AB_2533051
Vectashield	Vector Laboratories	Catalog #H-1000; RRID: AB_2336789
Deposited data		
Data (tracked behavior and connectomics)	This paper	https://www.dropbox.com/scl/fo/qbjrmhzd2lf1c6w6bdaej/AEjIfWnHWTMdy1V8mGB8JDY?rlkey=r3uncl0h35za4d87q7upeg6ip&st=lqqm9dvk&dl=0
Experimental models: organisms/strains		
*D. melanogaster*: w[1118]; P(y[ + t7.7] w[ + mC] = R33E02-p65.AD)attP40	Bloomington Drosophila Stock Center	BDSC: 70189; FlyBase: FBst0070189
*D. melanogaster*: w[1118]; P(y[ + t7.7] w[ + mC] = R47B03-GAL4.DBD)attP2	Bloomington Drosophila Stock Center	BDSC: 68657; FlyBase: FBst0068657
*D. melanogaster*: w[1118]; P(y[ + t7.7] w[ + mC] = R22D03-p65.AD)attP40	Bloomington Drosophila Stock Center	BDSC: 69869; FlyBase: FBst0069869
*D. melanogaster*: w[1118]; P(y[ + t7.7] w[ + mC] = R20E08-GAL4.DBD)attP2	Bloomington Drosophila Stock Center	BDSC: 69771; FlyBase: FBst0026818
*D. melanogaster*: w[1118]; P(y[ + t7.7] w[ + mC] = R47B03-p65.AD)attP40/CyO; P{y[ + t7.7] w[ + mC] = R30H02-GAL4.DBD)attP2/TM6B, Tb[1]	Bloomington Drosophila Stock Center	BDSC: 86709; FlyBase: FBst0086709
*D. melanogaster*: w[1118]; P(y[ + t7.7] w[ + mC] = VT060077-p65.AD)attP40; P(y[ + t7.7] w[ + mC] = VT029317-GAL4.DBD)attP2/TM6B, Tb[1]	Bloomington Drosophila Stock Center	BDSC: 86634; FlyBase: FBst0086634
*D. melanogaster*: w[1118] P(y[ + t7.7] w[ + mC] = 20XUAS-IVS-CsChrimson.mVenus)attP18	Bloomington Drosophila Stock Center	BDSC: 55134; FlyBase: FBst0055134
*D. melanogaster*: w[1118] P(y[ + t7.7] w[ + mC] = hs-FLPG5.PEST)attP3; PBac(y[ + mDint2] w[ + mC] = 10xUAS(FRT.stop)myr::smGdP-HA)VK00005 P(y[ + t7.7] w[ + mC] = 10xUAS(FRT.stop)myr::smGdP-V5-THS-10xUAS(FRT.stop)myr::smGdP-FLAG}su(Hw)attP1	Bloomington Drosophila Stock Center	BDSC: 64085; FlyBase: FBst0064085
Software and algorithms		
MATLAB r2022a	MathWorks	RRID: SCR_001622
Python v3.8.17	Anaconda (distributor)	RRID: SCR_008394
RStudio (R v4.1.1)	Posit	RRID: SCR_000432
DLC v2.3.5	Mathis et al., 2018; Nath et al., 2019	https://github.com/DeepLabCut/DeepLabCut RRID: SCR_021391
Natverse and other connectome-related packages	Bates et al., 2020	https://natverse.org/
JAABA	Kabra et al., 2013	https://jaaba.sourceforge.net/
Psychtoolbox 3.0	Brainard and Vision, 1997; Pelli, 1997; Kleiner et al., 2007	http://psychtoolbox.org/
DMD acquisition code and DLT triangulation GUI	This paper	https://github.com/bhandawatlab/DMDOptogeneticsExperiments
Analysis code and algorithms (behavior analysis, and connectomics)	This paper	https://github.com/bhandawatlab/2023_CL062_Tao_Bhandawat

### Experimental model and subject details

Flies were raised in 50 ml bottles of glucose media with ∼150 progeny/bottle (Archon Scientific D2). The number of progenies was controlled by the number of parent flies and the time parents were left on the food. A small sprinkle of active dry yeast was scattered on each bottle after removing the parents (1–3 d) to enrich the larvae diet. Bottles were placed in incubators set at 25°C (60% humidity) on a 12 h dark/12 h light cycle. Newly eclosed flies were raised in mixed 4 male + 4 female groups on 10 ml vials of D2 glucose media for control experiments. Retinal flies were raised with the same number of flies and put on food containing all-*trans*-retinal (0.02% by weight retinal) for optogenetic experiments. All vials were wrapped with aluminum foil to prevent retinal degradation and to keep conditions similar between retinal and control flies. Experiments were performed on flies 4 d after eclosion. Experimental flies were anesthetized on ice prior to placing them into the behavioral arenas.

### Freely walking behavior (Figs. 1–5, 10, 11)

#### Experimental setup

Behavioral experiments were performed in a setup similar to the one previously used in the lab (Chun et al., 2021). Flies were briefly immobilized using ice and placed onto a No. 1 coverslip. A 20 × 10 × 10 mm rectangular box made from cut microscope slides was secured over the fly using tape before the entire arena was held horizontally over a 45° mirror using clamps. The entire behavioral setup is enclosed in a Styrofoam box, and a blackout curtain was draped over the box. The temperature surrounding the behavioral arena is maintained at 25°C (±1 range) using a heating blanket (Oven Industries 5R1-013). Flies were given a 30 min acclimation period prior to the start of the experiment. We performed 15 trials per fly with a 5 min rest period in between trials. Each trial lasted 30.5 s with a 617 nm red light (Thorlabs M617L3) triggered to turn on between the 0.5 s and 15.5 s mark. The light intensity was measured at 8.9 mW/cm^2^. Videos were captured at 100 Hz using a camera (Basler acA1920-150um) and focused with a 35 mm lens (Edmund Optics 67-716). An infrared filter (Hoya IR76N) was placed at the end of the lens. The arena was lit using an 850 nm infrared light source (Thorlabs M850LP1).

#### Preprocessing of behavioral videos

The single-camera videos captured in the mirror chamber were split into a side view and a bottom view. For each view, we calculated the background by taking the mean of each pixel across a subsample of frames. Since there are trials where the fly stays in a single place, we then took the overall background as the mean of the backgrounds calculated across all trials for a given fly. After background subtraction, we calculated the log coefficient of variation (LCV) for each pixel as follows:

$$\mathrm{LCV}=\log_{10}\;\left(\frac{\sigma }{\mu }\right).$$

The foreground was defined as pixels with both an LCV of >0.25 and a log standard deviation (σ) of <0.45. We then applied built-in MATLAB functions to perform contrast-limited adaptive histogram equalization on the foreground images followed by image sharpening (Zuiderveld, 1994). Finally, the noise pixels were added back to the processed foreground image to limit the loss of information from pixels containing parts of the fly that are improperly classified as noise.

#### Detection of body parts using DeepLabCut (DLC)

The head, thorax, abdomen, and left and right wingtips were detected using DLC (Mathis et al., 2018). We used the inbuilt *k*-means clustering to pick out unique frames and manually annotated the body parts in each frame. We trained a single Resnet-50 (Park et al., 2016) for all corresponding cameras/views and both male and female flies. Prior to training, images were augmented using imgaug (A. B. Jung et al., 2020). After each round of training, the network with the lowest mean average Euclidean error (MAE) in the testing dataset was applied to full videos, and frames where tracked features had a confidence <0.8 were extracted. We then used *k*-means clustering on these low-confidence frames to select the next round of refinement frames. The final network was trained with a 702 and 37 image training/testing set (95% training set) and had an MAE of 4.74 pixels (100.68 µm).

#### Triangulation

A micromanipulator was used to move a fluorescent microbead fixed at the end of a pulled glass micropipette. A custom MATLAB graphical user interface (GUI) was created to capture images from both the side and bottom view of the microbead at 72 positions in a box in XYZ space and to perform semiautomated detection of the microbeads. We performed triangulation using direct linear transformation (DLT). The DLT root mean squared error was 63.34 mm.

#### Definition of observables and actions

Using the tracked head, thorax, abdomen tip, and left and right wingtips, we calculated the fly's speed, elevation angle, left-wing pitch, and right-wing pitch. We calculated the speed as the displacement over time of the tracked thorax position. To calculate the left (and right) wing extension angle (θ), we first defined the body (head–thorax–abdomen) vector as the linear least squares vector in the direction from the abdomen to the head. The wing extension angle (θ) was then defined as the angle between the negative body vector $\left(\overset{⇀}{v}\right)$ and the thorax wingtip vector $\left(\overset{⇀}{u}\right)$ as follows:

$$\theta =\left|\mathrm{atan}2\left(\frac{(\overset{⇀}{v}\times \overset{⇀}{u})\cdot \overset{⇀}{n}}{\overset{⇀}{v}\cdot \overset{⇀}{u}},\overset{⇀}{v}\cdot \overset{⇀}{u}\right)\right|.$$

The elevation angle was defined as the angle between the body vector and the closest surface the fly is standing on. This angle is calculated using the same formulation as the wing extension angle. The closest surface that the fly is standing on is determined by first calculating the normal vector of each wall using the third column of the right singular matrix after performing singular value decomposition on the wall plane centered at zero. Then, the distance to any given wall was calculated as the mean perpendicular distance between each endpoint (perpendicular projection of the head and abdomen positions onto the body axis) of the body axis and the wall.

To calculate the wing elevation angle, we first defined the plane formed by the tracked head, thorax, and abdomen points as the frontal plane with the normal vector pointing to the fly's left side as ${\overset{⇀}{n}}_{f}$. We can then project the wings and abdomen into the frontal plane as follows:

$$P_{\mathrm{projection}}=P-\frac{P\cdot {\overset{⇀}{n}}_{f}}{{\left\|{\overset{⇀}{n}}_{f}\right\|}^{2}}*{\overset{⇀}{n}}_{f},$$

where P is the wings or abdomen point and $P_{\mathrm{projection}}$ is the projection of that point on the plane. The wing elevation angles are then the angle between each wing projection to the abdomen projection in the frontal plane.

To calculate the wing azimuth angle, we defined the medial plane as the plane perpendicular to the frontal plane, coincident with the body vector and with the normal vector $\left({\overset{⇀}{n}}_{m}\right)$ facing upward. This was done by using the perpendicular projection of the head and abdomen positions onto the body axis, as well as another point defined by adding ${\overset{⇀}{n}}_{f}$ to the head projection on the body axis to define the plane. We can then project the wings and abdomen into this medial plane using the same formula as above but replacing ${\overset{⇀}{n}}_{f}$ with ${\overset{⇀}{n}}_{m}$. The wing azimuth angles are the angle between each wing projection to the abdomen projection in the medial plane.

**Figure 1.**
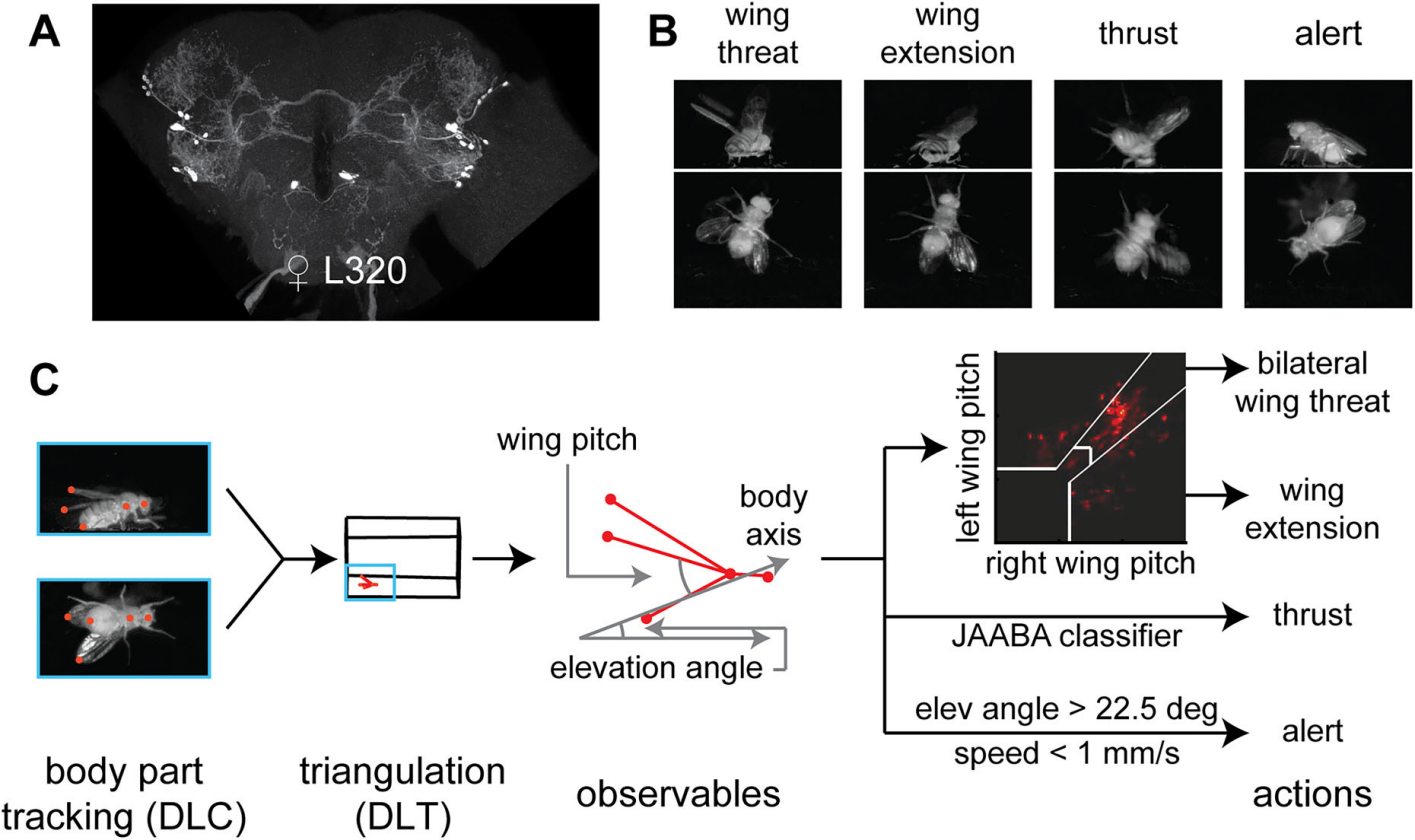
A framework for identification of actions driven by a small number of neurons. ***A***, Maximum intensity projection image of the female L320 brain (L320 > UASChrimson). ***B***, Example images of female flies displaying wing threat, wing extension, thrusting, and holding an alert posture when L320 neurons are optogenetically activated (L320 > UASChrimson). ***C***, Analysis pipeline. The head, thorax, abdomen, and two wingtips were tracked for two views using DLC. These body parts were 3D triangulated using DLT. Four observables (left/right wing pitch, elevation angle, speed) were calculated based on tracked body parts. Wing threat, wing extension, and alert stance were assigned based on thresholded observables. Thrusting was found using JAABA (see Materials and Methods and Figs. 2, 3).

**Figure 2.**
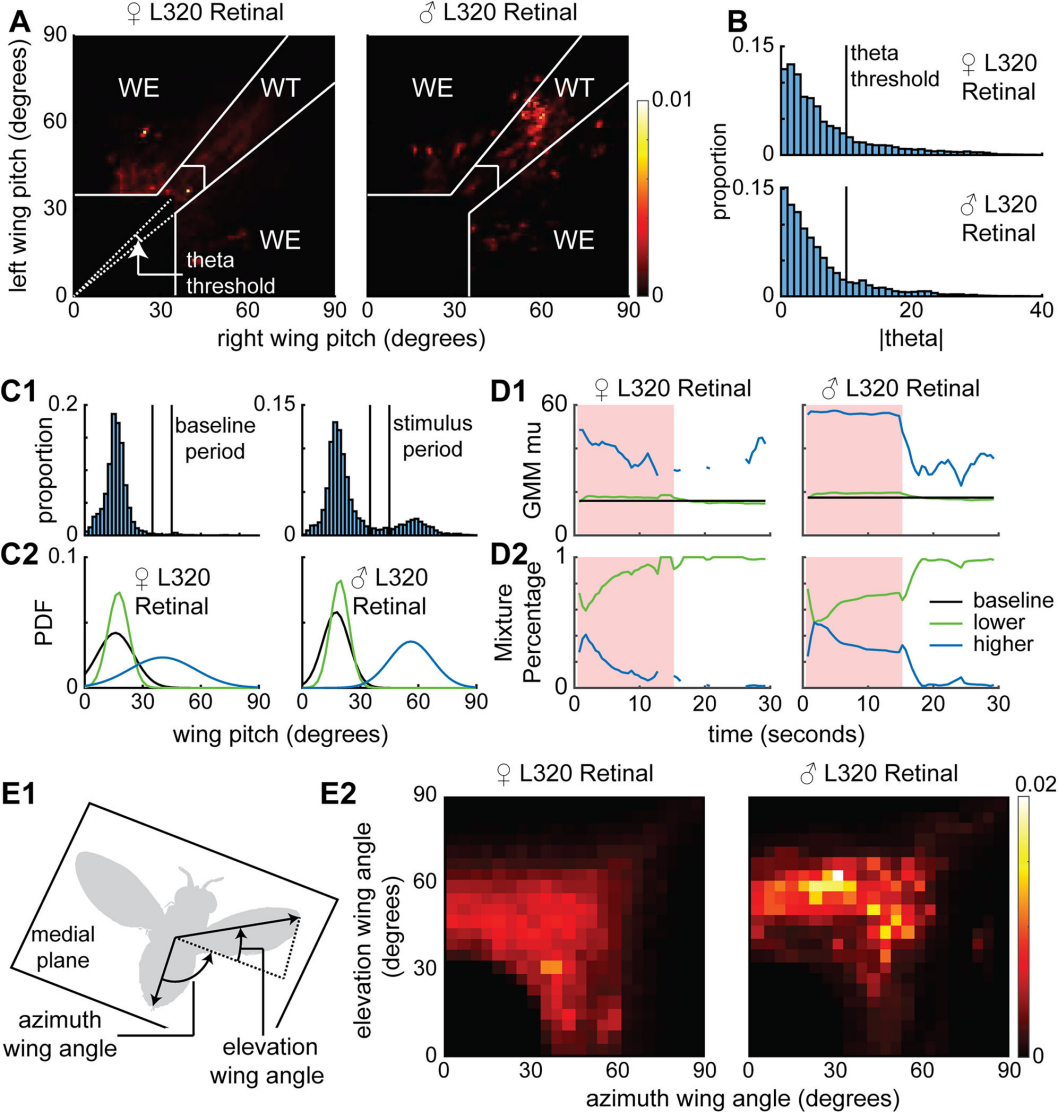
Wing threat can be distinguished from wing extension by comparing the elevation of the two wings. ***A***, WT, wing threat, and WE, wing extension, can be distinguished based on the wing pitch. WT occurs when either wing pitch is >45° with the wing that is less elevated is less elevated by <10° (the theta threshold). ***B***, The theta threshold was determined based on the distribution of the absolute angle the right- and left-wing pitch makes from the identity line (left pitch = right pitch). ***C*1**, Probability distributions of wing pitch for male and female L320 > UASChrimson flies before the stimulus period (left) and during the stimulus period (right) follow a roughly Gaussian and two-component Gaussian mixture distribution, respectively (***C*2**). The two black lines represent the threshold used in Figure 1 analysis and in panel ***A***. ***D***, Two-component GMM fits to 1.5 s (1 s overlap) sliding window wing pitch for female (left) and male (right) flies (see Materials and Methods). ***D*1**, The means of the GMM fits with the baseline being Gaussian fit to the before light on period shows that flies display both a higher wing pitch and a lower wing pitch throughout the stimulus period. ***D*2**, The mixing proportions of the two Gaussians in the GMM shows a faster decrease for female flies than for male flies. ***E***, The majority of wing behavior is a combination of an increase in both azimuth and elevation wing angle. ***E*1**, Schematic illustrating elevation and azimuth wing angle. The medial plane is defined as the plane that is orthogonal to the head–thorax–abdomen (frontal) plane and contains the body axis (see Materials and Methods). ***E*2**, Distribution of elevation and azimuth wing angles for female and male L320 > UASChrimson flies during wing threat and wing extension.

Wing threat and extension were defined based on wing pitches (Figs. 1*C*, 2*A*,*B*). First, a wing offset angle, which represents the extent that one wing is extended more than the other, is defined as the absolute angle between the line of unity (left pitch = right pitch) and the (*x* = left pitch; *y* = right pitch) vector. Wing threat was defined as when either the left- or right-wing pitch is >45° and the wing offset angle is <10°. Wing extension was defined as when either wing has a pitch > 35° and the wing offset angle is > 10°. A third region of low-wing threat was defined, but not characterized in this study. This region is defined as either wing having a pitch >35° and <45°, and the wing offset was <10°. The alert stance was defined by when the speed (thorax displacement) is <1 mm/s and the elevation angle was >22.5°. Thrusts were classified using a single JAABA classifier for both males and females with tracked observables (Kabra et al., 2013). We validated the performance of the classifier against manual annotations using 10-fold cross-validation and found 97.5% true positive and 96.9% true negative.

#### Analysis and statistical testing

Following DLC tracking, we marked tracked body parts with <70% confidence. Since two views were necessary for triangulation, the body parts were not triangulated for these instances and set to NaN. For continuous bouts of NaNs lasting <500 ms, we performed linear interpolation of the corresponding body part. When defining observables and actions, we set instances where dependent body parts were not triangulated or interpolated to NaN. For instance, if the side view of the left wing was marked as NaN at a particular time point, then the left-wing pitch, wing threat, and wing extension for that time point were set to NaN. These periods of NaNs are grayed out in the ethogram figures.

All ethograms as well as the observable traces were not smoothed. All statistical tests of actions over time were performed as follows. First, for each fly, we calculated the proportion of trials that the fly is performing the given action at each time point. This was smoothed by a 250 ms moving mean filter. To compare between two groups of flies, we looked at 500 ms nonoverlapping time bins (i.e., 0–0.5 s, 0.5–1 s, etc.) and averaged each fly's proportion over the time bin. We then calculated the significance using a Wilcoxon rank-sum test for each time bin using a significance cutoff of 0.01.

Latency to the first action bout was defined as the time from the light onset to the start of the first bout of each action within a trial (Fig. 5*A*2). The first bout was defined as the first bout that begins after the stimulus onset and lasts >30 ms.

During optogenetic stimulation, flies will keep their wings slightly ajar even when not performing wing threat or extension. As a first pass illustration of this, we fit Gaussian mixture models (GMM) to the wing pitch (Fig. 2*D*). We first fit a single Gaussian to the wing pitch in the 0.5 s period prior to the light onset. We then fit a two-component GMM to 1.5 s (1 s overlapping) sliding window wing pitches. Time windows where the Bayesian information criterion of the two-component GMM is higher than that of a single Gaussian fit or where the difference in the mean of the two Gaussians in the two-component GMM fit was <5° were fit using a single Gaussian. This analysis showed that:

1. There is a lower wing pitch distribution with an ever slightly higher mean than the baseline.
2. Female flies showed faster habituation of wing pitch over the stimulus period.

### DMD optogenetic experiments (Figs. 7–9, 12)

#### Experimental setup

Flies were immobilized using ice before being placed into a holder cut from a piece of aluminum foil. The head was stabilized using UV glue with the posterior brain facing upward. After submersion in external saline (103 mmol/L NaCl, 5 mmol/L KCl, 5 mmol/L Tris, 10 mmol/L glucose, 26 mmol/L NaHCO_3_, 1 mmol/L NaH_2_PO_4_, 1.5 mmol/L CaCl_2_, 4 mmol/L MgCl_2_), osmolarity and pH adjusted to 270–285 mOsm and 7.1–7.4, respectively, and bubbled with 95% O_2_/5% CO_2_, the cuticle was removed using forceps to expose the brain. A 20×/0.5 water immersion objective (Olympus Life Science UMPLFLN20XW) was used to visualize the brain. To activate different subpopulations of L320 neurons, we used a digital micromirror device (DMD) projector system (WINTECH PRO 4500) to project stimulus patterns through the objective to the neurons of interest. A 700 mm projection lens (comes with the DMD) was placed at the output of the DMD. The light then passes through an achromatic doublet (Thorlabs AC254-035-A-ML) to correct for chromatic aberrations. Next, a neutral density filter (Thorlabs NE13A-A) was placed in the light path to lower the intensity of stimulation. The achromatic doublet and neutral density filter were attached to the 1× camera adaptor for a dual port (Olympus U-DP/U-DP1XC) enclosing a 50R/50T beam splitter (Edmund Optics 35-944). A schematic of the setup is shown in Figure 7*A*.

Neurons were visualized using a CMOS camera (Hamamatsu Photonics Orca Flash 4.0). Prior to the start of the experiment, we took a *z*-stack of the fly brain to visualize the positions of neuron clusters expressing mVenus GFP. A custom GUI was made to define the circular stimulus ROIs (*x*, *y*, *z* position and radius) and create a stimulus train using precalibrated light intensity curves for user-specified wavelengths. Red light (617 nm) was used for Chrimson activation. Psychtoolbox (Brainard and Vision, 1997) was hardware triggered using a national instrument data acquisition system to present the stimulus train during experiments (NI USB-6363, 782258-01). The behavior was captured at 25 Hz using three video cameras (2× Basler aCA800-510uc and 1× Basler acA1920-150um) and focused with 0.5×, 94 mm lens (Infinity Photo-Optical InfiniStix 194050). The fly was illuminated with two infrared 850 nm light sources (Lorex vq2121).

The transformation between the projector coordinates to CMOS camera pixel coordinates was calibrated using an 8 × 8 grid of evenly spaced white dots in the projector space. These dots were mapped to positions in the camera space using an affine transformation. The light intensity was calibrated by projecting a small circle in the center of the CMOS camera view with a stimulus train consisting of increasing normalized projector power output, while a light meter (Thorlabs S121C and PM100USB) was placed underneath the objective to record the resulting light intensity. A No. 1 coverslip was placed on top of the light meter to immerse the 20× water immersion objective in external saline, and the objective was focused using a checkerboard pattern.

#### Detection of observables using DLC and triangulation

The head, thorax, first four strips on the abdomen, abdomen tip, and left and right wingtips in the three camera views were tracked using a single Resnet-50 model in DLC. The frame selection and training protocol were the same as that used for the freely walking DLC model. The final network was trained on 544 and 96 image training/testing sets (85% training set) and had an MAE of 4.03 pixels (38.69 µm). Calibration was performed for retinal-fed and nonretinal (control)-fed experiments separately since the cameras were moved in between the days when the two sets of experiments were performed. We calibrated the DLT triangulation using 108 and 95 positions for the retinal-fed and control experiments, respectively (we placed a microbead in 125 positions in a box in XYZ space, but not all positions were visible in at least two camera views). The DLT root mean squared error was 16.31 µm for retinal-fed flies and 48.53 µm for control flies.

#### Definition of observables and actions

While we tracked nine body parts, only the thorax, first stripe, and left/right wingtips were necessary to calculate wing pitch angles and wingspan. The wing pitch angles were calculated in the same manner as the mirror chamber rig. The only difference is that the $\overset{⇀}{v}$ vector is defined as the vector from the thorax to the first stripe on the fly abdomen. The wingspan was calculated as the distance between the wingtips. The wing pitch angle(s) and wingspan were baseline subtracted by the mean value during the 10 s prior to each light stimulation period.

#### Analysis and statistical testing

The first peak in wingspan (Fig. 9) was defined as the first peak in the baseline-subtracted wingspan that is greater than 0.22 mm for males and 0.25 for females. A smaller threshold was chosen for males since female wings are longer and, as a result, produce a larger wingspan. The ratio in the maximum male/female wingspan in the freely walking chamber was ∼0.88.

Figures 9, *B* and *C*, and 12, *C* and *D*, were analyzed using estimation methods to calculate mean, mean differences, and confidence intervals using a MATLAB toolbox (Claridge-Chang and Assam, 2016; Ho et al., 2019; Suver et al., 2019). Scatterplots show individual data points, and corresponding error bars show mean and bootstrapped 95% confidence interval (resampled 10,000 times, bias-corrected, and accelerated). The 95% confidence interval for differences between means was calculated using the same bootstrapping methods. *P* values were further generated using either Wilcoxon rank-sum or Wilcoxon signed-rank tests. An exact table of *p* values for Figures 8, *D* and *E*, and 12, *C and D*, can be found in Table 2.

**Table 2.** *P* values and *W* values

Figure 8*D*	Latency to first wing peak				
	Two-sided Wilcoxon rank-sum test				
Female (*p* values)	Intensity (mW/cm^2^)	0.5	1	2	4
	0.5	NaN	0.0156	0.0156	0.0469
	1	NaN	NaN	0.0313	0.2031
	2	NaN	NaN	NaN	0.7813
	4	NaN	NaN	NaN	NaN
Test *W* values	Intensity (mW/cm^2^)	0.5	1	2	4
	0.5	NaN	35	28	26
	1	NaN	NaN	21	22
	2	NaN	NaN	NaN	16
	4	NaN	NaN	NaN	NaN
Male (*p* values)	Intensity (mW/cm^2^)	0.5	1	2	4
	0.5	NaN	0.2262	0.0883	0.1817
	1	NaN	NaN	0.1496	0.1932
	2	NaN	NaN	NaN	0.7191
	4	NaN	NaN	NaN	NaN
Test *W* values	Intensity (mW/cm^2^)	0.5	1	2	4
	0.5	NaN	80	72.5	75
	1	NaN	NaN	374.5	393
	2	NaN	NaN	NaN	257.5
	4	NaN	NaN	NaN	NaN
Figure 8*E*	Change in wing baseline				
	Two-sided Wilcoxon signed-rank test				
Female (*p* values)	Intensity (mW/cm^2^)	0.5	1	2	4
	0.5	NaN	0.5195	0.1475	0.7646
	1	NaN	NaN	0.0244	0.8311
	2	NaN	NaN	NaN	0.5771
	4	NaN	NaN	NaN	NaN
Test *W* values	Intensity (mW/cm^2^)	0.5	1	2	4
	0.5	NaN	41	16	37
	1	NaN	NaN	8	30
	2	NaN	NaN	NaN	40
	4	NaN	NaN	NaN	NaN
Male (*p* values)	Intensity (mW/cm^2^)	0.5	1	2	4
	0.5	NaN	0.2692	0.8489	0.0115
	1	NaN	NaN	0.9292	0.0063
	2	NaN	NaN	NaN	0.0409
	4	NaN	NaN	NaN	NaN
Test *W* values	Intensity (mW/cm^2^)	0.5	1	2	4
	0.5	NaN	132	183	275
	1	NaN	NaN	172	283
	2	NaN	NaN	NaN	256
	4	NaN	NaN	NaN	NaN
Figure 12*C*	Ipsi versus contra wing pitch change				
	Two-sided Wilcoxon signed-rank test				
(*p* values)	Intensity (mW/cm^2^)	first 2 s	last 2 s	full 5 s	
	Both	0.0000032	0.0000112	0.0000003	
	Left	0.0000715	0.0208000	0.0006397	
	Right	0.0129000	0.0001343	0.0001175	
Test *W* values	Intensity (mW/cm^2^)	first 2 s	last 2 s	full 5 s	
	Both	3,335	3,267	3,456	
	Left	904	752	853	
	Right	768	890	893	
Figure 12*D*	Bilateral versus sum of ipsilateral and contralateral wing pitch change				
	Two-sided Wilcoxon signed-rank test				
(*p* values)	Intensity (mW/cm^2^)	first 2 s	last 2 s	full 5 s	
	Both	0.0000955	0.0001314	0.0000078	
	Left	0.0058000	0.1024000	0.0111000	
	Right	0.0081000	0.0001907	0.0001229	
Test *W* values	Intensity (mW/cm^2^)	first 2 s	last 2 s	full 5 s	
	Both	3,141	3,121	3,287	
	Left	793	690	773	
	Right	783	882	892	

Table is referenced from figure legend.

### Identifying neurons labeled by L320 (Fig. 6)

#### Immunohistochemistry and imaging

Dissections and immunohistochemistry were carried out on Chrimson flies 3–5 d after eclosion. For Multicolor Flipout (MCFO) experiments, 3-d-old flies were heat shocked at 37°C for 25–30 min. Dissections and immunohistochemistry were carried out 2–3 d after the heat shock. Flies were dissected in PBS, fixed in 2% paraformaldehyde for 55 min, blocked for 1.5 h using 5% NGS, incubated in primary antibody in 5% NGS for at least overnight, incubated in secondary antibody in 5% NGS for at least two overnights, and mounted using Vectashield (Vector Laboratories H-1000). After each step, the tissue was rinsed with PBS followed by 3 × 20 min wash using 0.5% Triton-X in PBS. Antibody incubation was performed on a 2D nutating shaker at 4°C. All other steps were performed on a 3D nutator at room temperature. The following primary antibodies were used: chicken anti-GFP (1:400 Abcam ab13970) and mouse anti-NC82 (1:20 Developmental Studies Hybridoma Bank). The following secondary antibodies were used: Alexa Fluor 488 anti-chicken (1:600 Invitrogen A11039) and Alexa Fluor 633 anti-mouse (1:400 Invitrogen A21050). The following conjugate antibodies were used for MCFO experiments: Dylight 550 anti-FLAG (1:200 Invitrogen MA1-91878-D550) and Dylight 488 anti-HA (1:200 Invitrogen 26183-D488). Fluorescence images were acquired using a Zeiss LSM700 inverted microscope confocal microscope.

### Brain registration and neuron matching (Fig. 6)

For the identification of L320 neuron IDs, we performed MCFO on female brains. We used CMTK to register dissected female brains to the JFRC2 template brain space. After registration to the JFRC2 template, we performed neuron tracing using the simple neurite tracer in ImageJ (Arshadi et al., 2021). The traced neurons were transformed to the hemibrain space (JRCFIB2018F) via JFRC2010 and JRC2018F using the neuroanatomy toolbox (Bates et al., 2020; Bogovic et al., 2021). We then calculated a similarity score between transformed neurons to all hemibrain neurons using NBLAST (Costa et al., 2016). Hemibrain neurons with a NBLAST score of >2,500 were visually inspected to determine the most likely neuron match.

### Connectomics analysis (Figs. 13–15)

#### Choice of neuron IDs

We utilized Codex (Snapshot v783 October 2023 release) to obtain a list of neurons that are classified as CL062, pC1, aIPg, aSPg, and DNs (Matsliah et al., 2023). For CL062 neurons, we further stipulated that they were given the community label of AIP since that was the given name to these neurons in the paper that first characterized the CL062-split genetic line (Duistermars et al., 2018). A list of neuron IDs with their corresponding type and hemisphere can be found in Table 3. This list does not include all DNs, only the ones mentioned by name or with morphology shown in the figures.

**Table 3.** FAFB/Codex neuron table

Neuron ID	Type	Hemisphere
List of CL062, pC1, aIPg, aSPg neurons considered in the connectomics analysis. List of the DNs listed by name and/or whose structure is shown		
720575940626885124,720575940634371941,720575940608082990,720575940630037073,720575940637079667,720575940606687957,720575940614437462,720575940633205295	CL062 (AIP, AVLP_pr12)	Left
720575940628333788,720575940620424472,720575940635130905,720575940626869403,720575940632011420,720575940637319134,720575940624273395,720575940625194014,720575940616650589	CL062 (AIP, AVLP_pr12)	Right
720575940646310947	pC1a	Left
720575940625792698	pC1a	Right
720575940629401674	pC1b	Left
720575940619855296	pC1b	Right
720575940618984475	pC1c	Left
720575940629430978	pC1c	Right
720575940646518382	pC1d	Left
720575940620356545	pC1d	Right
720575940634866586	pC1e	Left
720575940618364480	pC1e	Right
720575940637281395,720575940612938645	aIPg1	Left
720575940625645128,720575940638776227	aIPg1	Right
720575940628599934,720575940625855004	aIPg2	Left
720575940612290986,720575940624780062,720575940604458720,720575940632525920,720575940640869795,720575940609834052,720575940626744838,720575940632809823, 720575940626044746,720575940636443828,720575940615641428,720575940634736538,720575940610898659,720575940618887021,720575940625384732,720575940637101375	aIPg3	Left
720575940633307729,720575940627589126,720575940617591687,720575940620431752,720575940634109326,720575940626255632,720575940625871132,720575940637165987, 720575940618376614,720575940620085670,720575940609970090,720575940605856689,720575940625952186,720575940619406661, 720575940623865417,720575940636747854,720575940629531351,720575940634418784,720575940610867683,720575940634248559,720575940611554034	aIPg3	Right
720575940627354562,720575940622752041,720575940623730634,720575940637282675,720575940617574229,720575940614081909,720575940640889176	aIPg4	Left
720575940633353327,720575940645931060,720575940628135675,720575940624211075,720575940622971911,720575940619859532,720575940651713782,720575940623766458,720575940617900123	aIPg4	Right
720575940629852163,720575940615109563,720575940630812663	aIPga	Left
720575940614310050,720575940626953063,720575940613518092,720575940630355537,720575940610754162,720575940611764066	aIPga	Right
720575940632821864,720575940634814315,720575940606411698,720575940630899560,720575940636004836	aSPg1	Left
720575940630245650,720575940608481860,720575940631434541	aSPg1	Right
720575940631877944,720575940630181463,720575940618995505,720575940624290303	aSPg2	Left
720575940611682533,720575940616592523,720575940627600719,720575940638890355,720575940639053146	aSPg2	Right
720575940627607192,720575940626754192,720575940633118637	aSPg3	Left
720575940615606634,720575940626220669,720575940627700554	aSPg3	Right
720575940624977847,720575940631499832	DNpe050 (CL341)	Left/right
720575940620582260,720575940630221509	DNp67 (pMP12)	Left/right
720575940629477675,720575940632296519	DNge079	Left/right
720575940624897516,720575940647029044	DNg105	Left/right
720575940623781127,720575940610505006	DNp103 (PVLP119)	Left/right
720575940622673860,720575940611644529	DNp06	Left/right

#### Determining equivalent connection weights

We utilized fafbseg to retrieve full adult fly brain (FAFB) reconstructions from the production version of FlyWire (queried May 2024) to perform all connectomics analysis (Zheng et al., 2018; Li et al., 2020; Bogovic et al., 2021; Buhmann et al., 2021; Dorkenwald et al., 2022). We set a cutoff of 10 synapses between any two neuron pairs for all connectivity analysis and considered the number of synapses between neurons as the edge weight between neurons. A threshold of 5–10 synapses is commonly applied in past studies using the connectome (Li et al., 2020; Schlegel et al., 2023; Eckstein et al., 2024). We chose a threshold of 10 because with five synapses, there were too many partners over two synapses making the computation very slow. We utilized a resistor circuit approach to model equivalent (total) weight between two neurons that considers indirect paths through any arbitrary number of intermediate neurons. Under this formulation, the edge weights are treated as the conductance of a resistor. Therefore, the path weight for a single path between two neurons that passes through multiple intermediate neurons (series) is the inverse of the sum of the inverses of the individual weights between neurons in the path. Meanwhile, the equivalent weight for all paths between two neurons is simply the sum of all the individual path weights. In this paper, we considered only direct paths or paths with up to one intermediate neuron.

We used fafbseg to retrieve neurotransmitter/modulator (NT for brevity) predictions for each postsynaptic site from FlyWire using a cleft score of 0 (Eckstein et al., 2024). These include GABA, acetylcholine, glutamate, octopamine, serotonin, and dopamine. The most confident NT was assigned to each synaptic site. The NT predictions between two neurons were defined as a six-dimensional vector representing the number of synapses. Given a two-layered path between two neurons (i.e., three nodes with two edges in series), there are 36 total permutations of path types (e.g., GABA → Ach, Ach → GABA, etc.). However, since almost all synaptic assignments for CL062, aIPg, and pC1 neurons were cholinergic, in practice, the number of likely permutations was at most 6. Following our definition of equivalent weights, the equivalent weight associated with any permutation of NT *i* to NT *j* is as follows:

$$W_{\mathrm{path}}^{i\to j}=\frac{W_{1}^{i}*W_{2}^{j}}{W_{1}+W_{2}},$$

where $W_{1}$ and $W_{2}$ are the total number of synapses (i.e., weight) associated with each edge of the path. $W_{1}^{i}$ and $W_{2}^{j}$ are the total number of synapses assigned to the *i*th and *j*th NT for the two path edges, respectively.

#### Clustering/PCA/SVM analysis

CL062 were clustered by their connections to DNs by utilizing cosine similarity. Starting from the matrix defining the equivalent weight from CL062 (rows) to DNs (columns), cosine similarity is performed by performing a Euclidean normalization on each row. Next, the cosine similarity is defined as the cosine of the angle between each pair of rows. After performing cosine similarity, we performed hierarchical agglomerative clustering to cluster the CL062 neurons into three clusters. For each cluster, we calculated the average equivalent weight for each CL062 neuron in the cluster to each DN (Fig. 7*F*). A threshold of 20 was set because this was an approximate elbow point in the distribution of equivalent weights. The same process was repeated for building the similarity matrix for all alPg and CL062 neurons based on connections to DNs (Fig. 8*A*).

Principal component analysis (PCA) was performed on Euclidean normalized equivalent weight matrix from CL062 and aIPg to all DNs. The first three PCs explain 19.48, 16.24, and 12.80% of the variance, respectively. The PC scores were scaled by dividing by the maximum absolute value of the first three PC scores and then multiplying by the maximum distance of the loadings for the first three PCs. This scaling allows for viewing the loadings and the PC score in a biplot. The first three PCs separated the left/right CL062 and aIPg neurons into different clusters. We created two planes using support vector machines (SVMs) in the PC space, one plane to differentiate based on hemisphere and another to differentiate based on aIPg versus CL062. We used 10-fold cross-validation to obtain a loss of 0.02 and 0 for the hemisphere and class support vectors, respectively. Both PCA and SVM were performed using standard MATLAB function. For this analysis, we only considered CL062 and aIPg neurons that made a total equivalent weight of at least 300 across all potential DNs. While the total effective weight across CL062/aIPg to DNs range from 0 to 5,900 (with many aIPg neurons weakly connected to DNs), we found the results of the PCA and SVM analysis (SV loss, angle between SVs, and the DNs highlighted in Fig. 14*B*,*C*) to be robust for thresholds up to 1,100—above which, the number of CL062 neurons being considered becomes too low.

## Results

### Activation of L320 neurons drives multiple aggressive behaviors in isolated male and female flies

**Figure 3.**
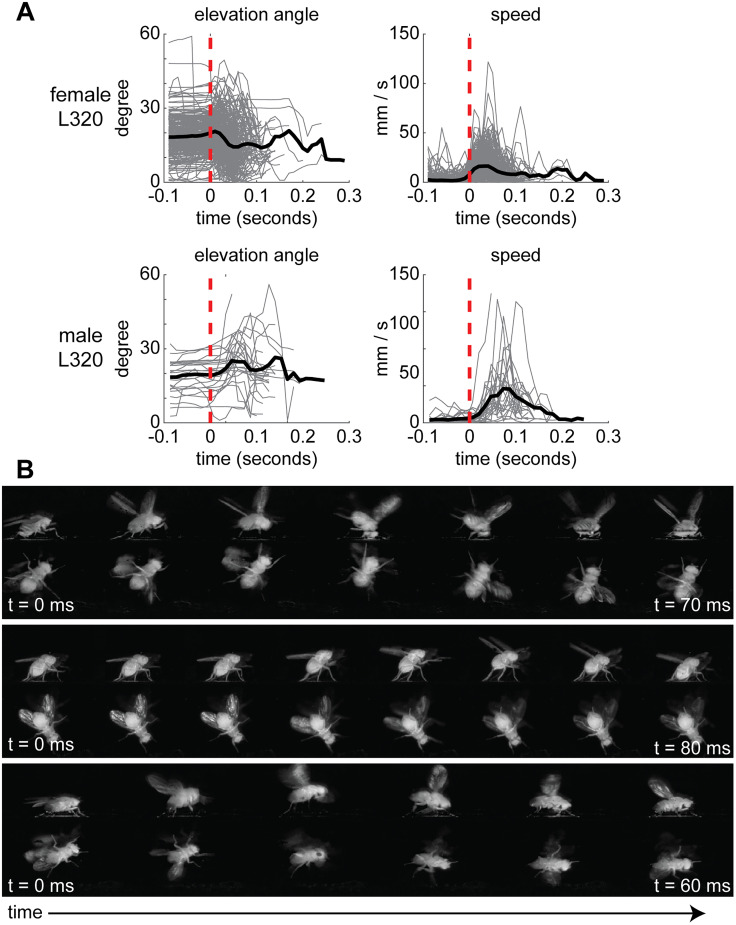
Thrust is a suite of related actions that can be identified using a combination of body elevation angle and speed. ***A***, Elevation angle and speed aligned by manually annotated bouts of high fence used as ground truth (*n* = 75 female trials; *n* = 60 male trials). Red dotted line indicates start of a thrust bout. Thrust is characterized by an increase in elevation angle as the forelegs are raised. At the end of the thrust, flies will snap their forelegs down and drive the body toward the ground, resulting in a drop in the elevation angle. ***B***, Three examples of different types of thrusting. Top, High-intensity female thrust. Middle, Low-intensity female thrust. Bottom, High-intensity male thrust.

In a behavioral screen on the collection of Janelia split-Gal4 collection of genetic lines (Dolan et al., 2019), we found that optogenetic activation of neurons using the red-shifted channelrhodopsin, CsChrimson (Klapoetke et al., 2014), in a split-Gal4 line, L320 (GMR33E02-AD ∩ GMR47B03-DBD; Fig. 1*A*), drives multiple aggressive actions including wing threat, wing extension, and thrusting, as well as holding an alert posture in both isolated male and female flies (Fig. 1*B*; Movies 1, 2). A hallmark of aggressive behavior—seen here (Fig. 1*B*, leftmost panel)—is that the wing movement is upward (Fig. 1*B*) as opposed to the sideways wing extension observed during courtship. Aggressive phenotypes elicited by optogenetic activation of the L320-split line can occur in isolation (i.e., without either the presence of any target of their aggression or resources that they typically fight over); this expression of aggression in the absence of a target is rare as most studies report aggressive actions only in the presence of a conspecific (Dankert et al., 2009; Certel and Kravitz, 2012; Kravitz and Fernandez, 2015). To quantify the behavior, we tracked five body parts of the fly—head, thorax, abdomen, and left/right wingtips—on each of the two camera views. The tracked body parts were triangulated to yield five features (Fig. 1*C*). The relationship between the observables obtained from these features and the four aggressive actions—wing threat, wing extension, thrusting, and alert stance—is shown in Figure 1*C* and described in detail in the next two figures and corresponding text (Figs. 2, 3).

First, we quantified the relationship between the positions of the two wings and wing extension and threat (Fig. 2). Based on previous work (Nilsen et al., 2004), we only quantified aggressive actions involving wings when the wings were pitched up. Even though it was noted in the previous work that wing threat could be unilateral or bilateral, both behaviors were classified as wing threat. In this study we distinguish between bilateral wing threat and unilateral wing extension. Bilateral wing threat occur when either wing is raised >45°, as long as the other wing is also raised at least 35° (Fig. 2*A*). Unilateral wing extensions occurred either when no wing was pitched >45° or the difference between wing pitches was >10°. Unlike wing threats—which last ∼1 s—wing extension can last much longer, and, therefore, we considered it as a separate behavior; the method used to distinguish between wing threat and wing extension is described in Figure 2*A–C*. We also show that the change in wing pitch is described by two separate behaviors—wing threat and wing extension—with the contribution of wing extension increasing with time (Fig. 2*C*,*D*). Using a Gaussian mixture model, we show that the wing threat decreases much faster in females. During bilateral wing threats and most wing extensions, flies raised their wings at a ∼45° elevation from the body plane; this suggests that these actions represent different forms of wing threat rather than horizontal wing extension exhibited during courtship (Fig. 2*E*). In addition, flies also keep their wings slightly ajar when not performing wing threat or extension (Movies 1, 2; Fig. 2*C*,*D*); here we focused on wing threat and wing extension in this study.

Next we identify thrusting using single JAABA classifier using movement speed and elevation angle (Fig. 3). Both speed and elevation angle are employed (Fig. 3*A*) because, at the start of a thrust, flies lift their front leg and pitch up resulting in an increase in elevation angle and speed. Afterward, flies push forward and snap down on their front legs often resulting in a lower-than-baseline elevation angle (Fig. 3). As in previous work (Nilsen et al., 2004), different thrust episodes varied in how the body is elevated and whether only the front legs are lifted off the ground or the other legs as well (Fig. 3*B*). There are also differences in how the body snaps back. Our classifier groups different forms of thrusting in a manner comparable with similar grouping together of thrust in previous studies (Nilsen et al., 2004). It is important to note that a similar action—where the fly raises the front part of its body and lunges down—in males is referred to in some studies as a lunge (Hoffmann, 1987). There is some ambiguity in how different authors define these actions (Asahina, 2017); we are following the convention outlined in Nilsen et al. (2004). Finally, we used a combination of speed and elevation angle thresholds to capture an alert stance when the flies stood still with their front legs straightened in an upward-pitched position (Fig. 1*C*). An alert stance may be a novel form of aggression that has not been reported (to our knowledge), likely because most work on aggression uses a single-camera view which is insufficient for detecting an alert pose. We also observed other behaviors such as retreat and take-off but have not quantified these behaviors in this study.

**Figure 4.**
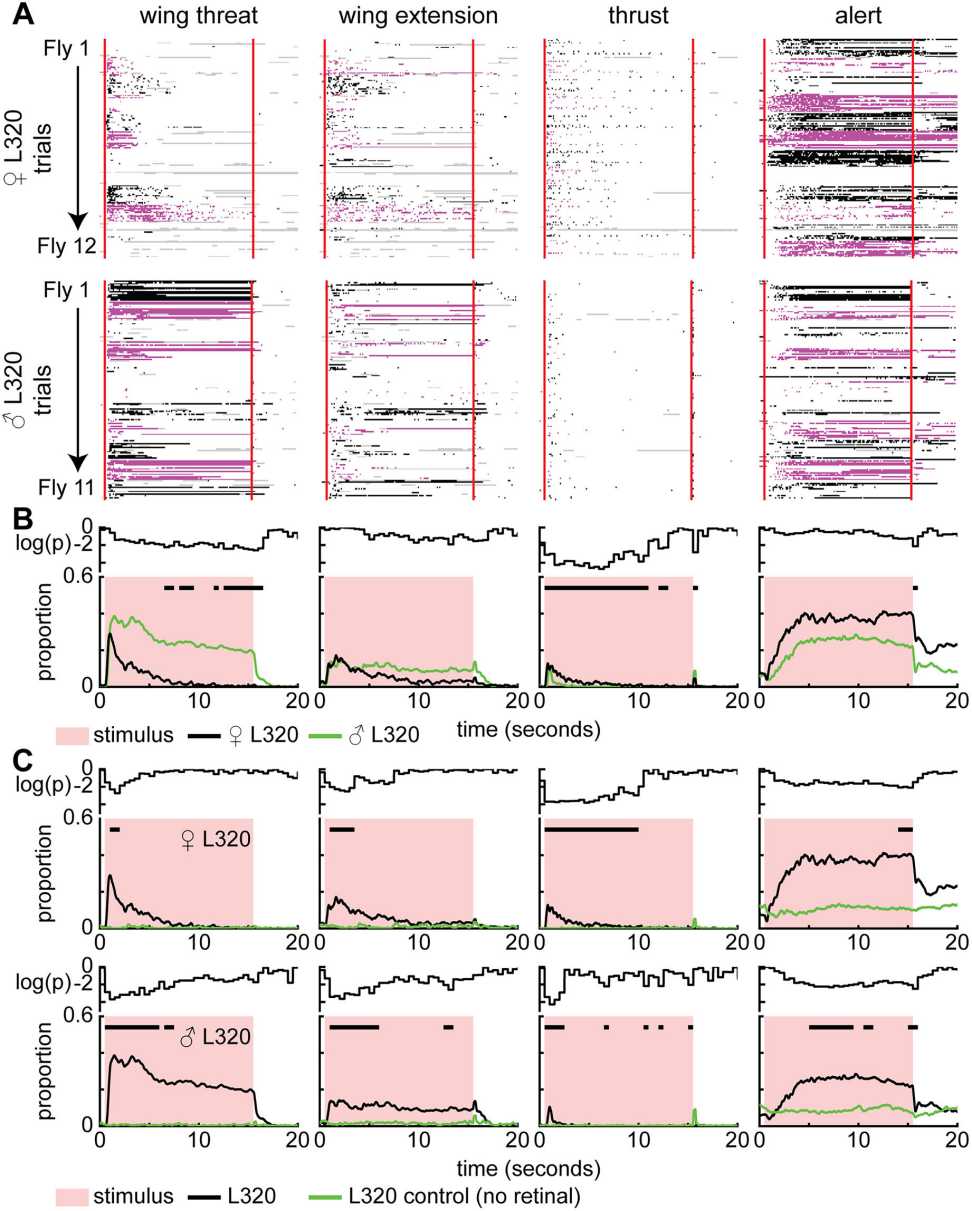
The activation of a small number of neurons drives sexually conserved set of aggressive actions and observables in the absence of sensory information. There is sexual dimorphism in the level of these actions. ***A***, Single-trial ethogram of actions for female and male L320 > UASChrimson flies. Trials grouped by flies (Fly 1, Trials 1–15; Fly 2, Trials 16–30; etc.). The red line indicates the start and end of optogenetic stimulation period, which lasts for 15 s. Alternating black and purple blocks of trials represent different flies. Gray areas indicate >500 ms bouts where there is low confidence in any of the tracked body parts used to identify the action (see Materials and Methods). ***B***, Proportion of all male and female trials where flies are performing each action. Black bars show time points where there is a significant difference between male and female flies (Wilcoxon rank-sum test *p* < 0.01; see Materials and Methods). An 8.9 mW/cm^2^ 617 nm light is turned on during the stimulus period (retinal fed, *n* = 12 female flies; *n* = 11 male flies; 15 trials/fly). ***C***, Top row, Proportion of trials where female L320 > UASChrimson flies not fed with retinal are performing each action (retinal fed, *n* = 12 flies; control, *n* = 8 flies; 15 trials/fly). Black bars show time points where there is a significant difference between retinal fed and control flies (Wilcoxon rank-sum test *p* < 0.01; see Materials and Methods). Bottom row, Same as top row, but for male L320 > UASChrimson flies (retinal fed, *n* = 11 flies; control, *n* = 11 flies; 15 trials/fly). For ***B*** and ***C***, the top row shows log10 of the *p* value.

Next, we characterized the temporal relationship between the four aggressive actions. Aggression is characterized by a complex structure consisting of recurring behavioral sequences (Chen et al., 2002; Nilsen et al., 2004; Hoopfer, 2016); we observe a similar complex behavioral structure when activating the L320 neurons (Fig. 4*A,B*). Aggressive actions are not observed in flies that are not fed retinal (Fig. 4*C*). There is considerable variability in the occurrence, latency, and persistence of each of the actions (Fig. 4). As an example, wing threat does not occur in any trial in one fly in our dataset and occurs in only some trials in another fly (Fig. 4*A*). Despite the moment-by-moment action being different across different trials and flies, the probability of observing a given action has a clear temporal progression. The probability of observing wing threat was highest shortly after the stimulus onset, decreased rapidly in females and slowly decreasing in males (Fig. 4*B*). Wing extension follows a similar trend to wing threat with males showing more persistence (Fig. 4*B*). Unlike wing threat and extension, males perform significantly fewer thrusts and exhibit faster habituation of this action (Fig. 4*B*). Wing-driven behavior appears to occur a greater proportion of the time compared with thrusts. However, this lower moment-by-moment proportion of flies performing thrusts is due to their transient nature (Fig. 4*B*). At the light offset, flies will sometimes perform thrusts or controlled jumps that are time-locked to stimulus-off (Fig. 4*B*). This results in an instantaneous peak in the proportion of flies performing thrusts (Fig. 4*B*). After a period of thrusting, flies often transition into an alert posture. Unlike other actions, this alert stance is persistent even after the stimulus has been turned off.

**Figure 5.**
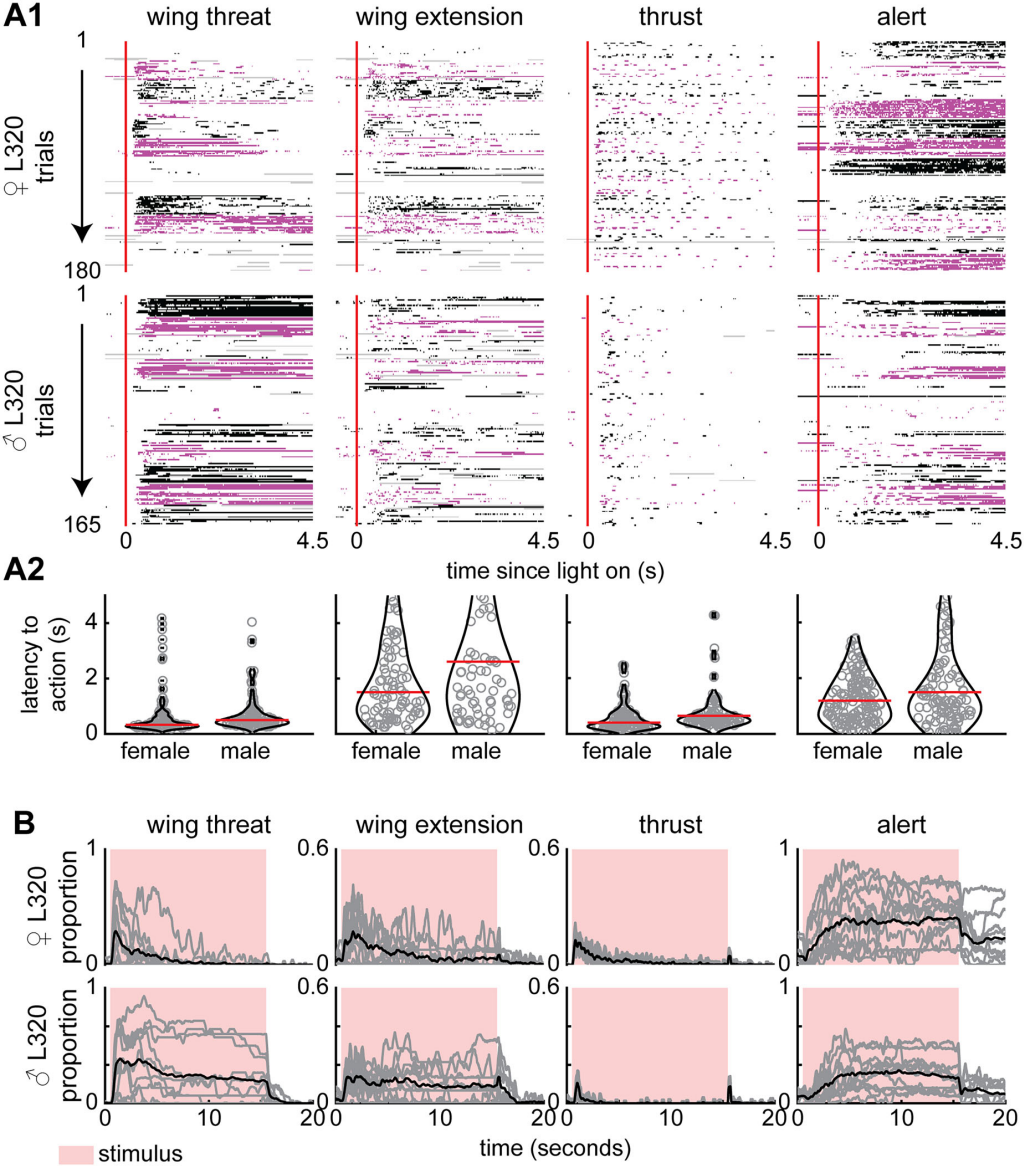
Optogenetic activation of L320 neurons drives action production at low latency. ***A*1**, Single-trial ethogram of actions for male and female L320 > UASChrimson flies for the first 5 s. Trials grouped by flies (Fly 1, Trials 1–15; Fly 2, Trials 16–30; etc.). The red line indicates the start of optogenetic stimulation period. Alternating black and purple blocks of trials represent different flies. Gray areas indicate frames where any of the tracked body parts used to identify the action had low tracking confidence (see Materials and Methods). ***A*2**, Latency from the stimulus onset to first instance of each action across all trials. Violin plots show distribution of latency. Red line shows median latency. ***B***, Proportion of trials that each male and female L320 > UASChrimson is performing each action as a function of time. Each gray line is a fly, and the black line is the average across all flies. An 8.9 mW/cm^2^ 617 nm light is turned on during the stimulus period (retinal fed, *n* = 12 female flies; *n* = 11 male flies; 15 trials/fly).

To better illustrate the structure of the behavior, we show ethograms of the first 4.5 s (Fig. 5*A*). There is an alternation between wing threat, wing extension, thrust, and alert state (Fig. 5*A*1). In addition, most thrusts occur simultaneously with a wing threat or extension. In most trials, wing threat is the first action and can occur in under 300 ms (Fig. 5*A*2), followed by thrust. Wing extensions and alert posture occurred with more delay. The differences in behavior between males and females are also clear. The wing threat is more persistent in males than in females; many males did not show any habituation (Fig. 5*B*).

### L320 labels four populations of neurons and a DN

**Figure 6.**
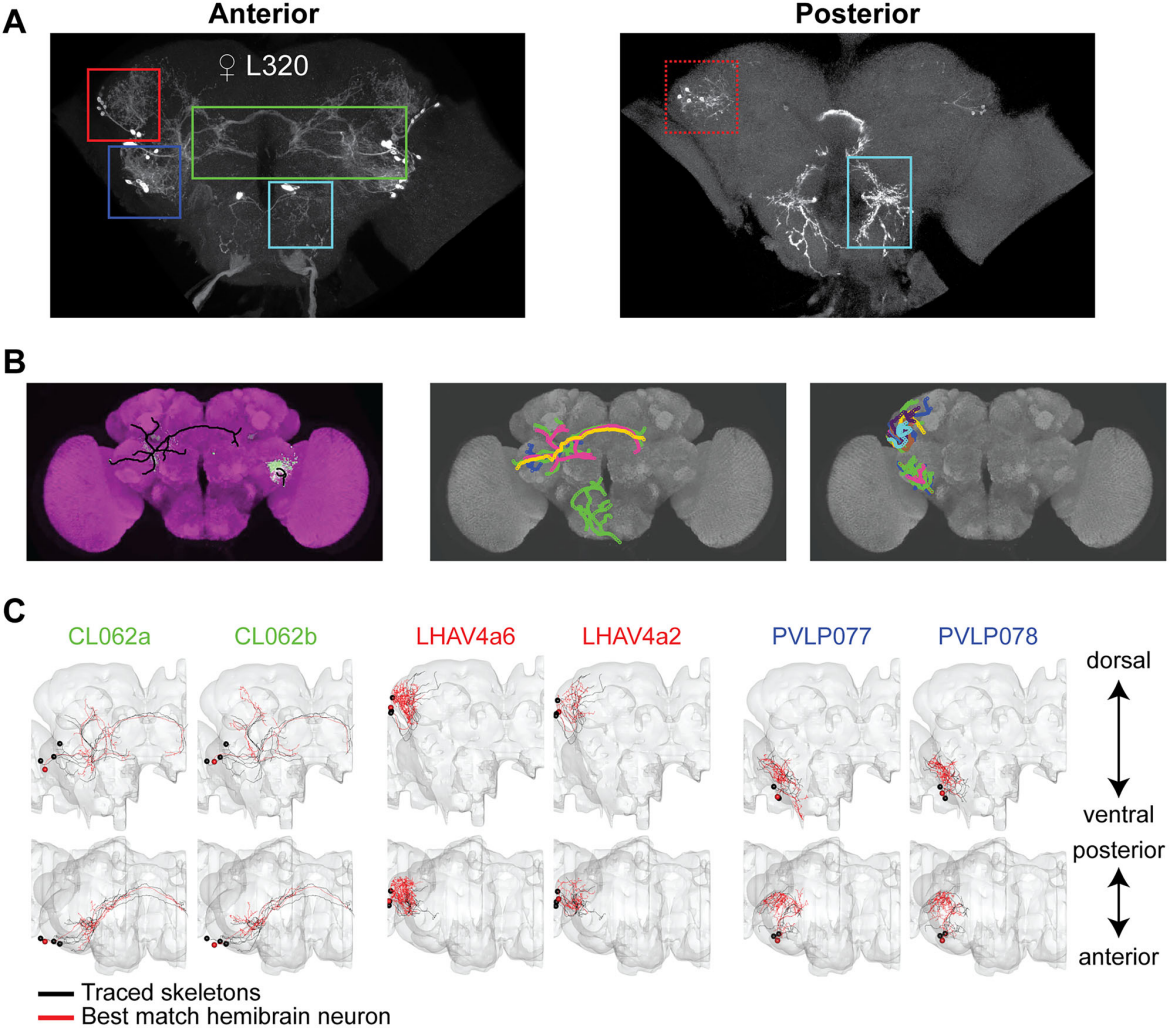
Neuron types labeled by L320. ***A***, Maximum projection image of female L320 in the anterior plane (left) and posterior plane (right). L320 labels two types of LH neurons (red and dashed red), one type of AVLP neuron (green), one type of posterior VLP (PVLP) neuron (blue), and a pair of DNs (cyan). ***B***, Left, Sample MCFO brain registered to JFRC2 with neurons in green and JFRC2 template brain in purple. The traced skeletons for neurons are shown in black. Middle, All skeletonized right hemisphere neurons in the AVLP and the single DN. Right, Same as middle panel, but for neurons in the anterior LH and PVLP. Each color represents a different neuron trace. ***C***, Top matched hemibrain classes for the AVLP (green), anterior LH (red), and PVLP (blue) clusters of neurons labeled by L320. Sample traced skeletons from MCFO are shown in black. The top matched hemibrain neurons based on Nblast and visual inspection are shown in red. We were unable to identify the DN in the hemibrain.

L320-split, despite being sparse, labels multiple populations of neurons (Fig. 6*A*). To identify these neurons, we utilized a morphology-matching algorithm called NBLAST to compare traced neurites of L320-split neurons with skeletons within a recently released female electron microscopy dataset called the hemibrain dataset (Costa et al., 2016; Scheffer et al., 2020). Because the neurite of different neuronal populations intersect, we used multicolor flip (MCFO) to stochastically label 1–3 L320 neurons in each fly brain so that we could trace them unambiguously (Fig. 6*B* for one example; Nern et al., 2015). The traced neurons were morphologically located in the lateral horn (LH), anterior ventrolateral protocerebrum (AVLP), posterior ventrolateral protocerebrum (PVLP) regions, as well as a single pair of DNs. We found that the anterior cluster of LH neurons, which formed a characteristic U-shaped neurite tract that first runs in the anterior–ventral direction before looping back in the posterior–dorsal direction and arborizes within the lateral portion of the LH, has a similar morphology to LHAV4a in the hemibrain (Fig. 6*C*). LHAV4a are structurally connected to projection neurons implicated in bilateral contrast sensing of *cis*-vaccenyl acetate (cVA; Taisz et al., 2023). cVA is the primary pheromone implicated in fly courtship and aggression (Kurtovic et al., 2007; L. Wang and Anderson, 2010; W. Liu et al., 2011). The posterior cluster of LH neurons was identified as LHPV6a1/3 in the hemibrain. The AVLP neurons have a similar morphology to the CL062 neurons since they both cross the midline along an anterior–posterior curve and form characteristic dorsal–ventral branches (Fig. 6*C*). These CL062 neurons have previously been shown to drive aggressive male threats (Duistermars et al., 2018); they are also referred to as AVLP_pr12 neurons (Baker et al., 2022). The PVLP neurons have a similar morphology to PVLP077/078 neurons due to their anterior–ventral to posterior–dorsal neurite track (Fig. 6*C*). Finally, none of the DNs in the hemibrain dataset matched the DN labeled by L320-split. We did not search for the DN in the FlyWire dataset further.

### Activation of CL062 neurons drives aggressive actions

**Figure 7.**
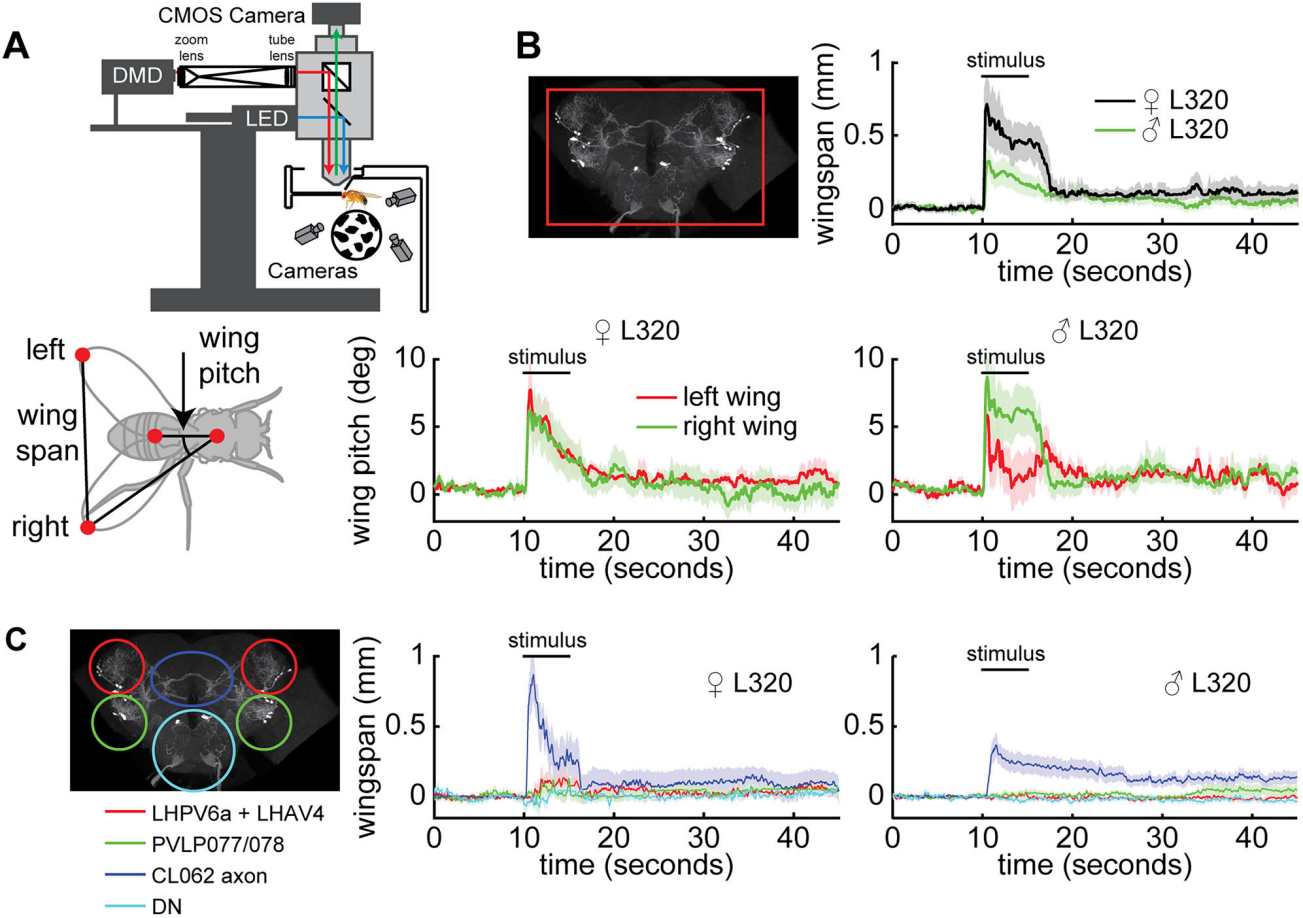
Optogenetic activation of a single population of neurons (CL062) drives wing threat in both females and males. ***A***, Experimental setup. Bottom, Top–down diagram of a fly illustrating left/right wing pitch and wingspan. ***B***, Optogenetic activation of all L320 neurons (L320 > UASChrimson) for males and females (females, *n* = 11 trials across 5 flies; males, *n* =  26 trials across 7 flies). The baseline-subtracted wingspan (top right) and wing pitches (bottom row) following optogenetic stimulation using a 2 mW/cm^2^ 617 nm light. ***C***, Spatially targeted optogenetic activation of each type of neuron labeled by L320 > UAS Chrimson. Left, Illustration of regions targeted. Middle and right panels show baseline-subtracted female and male wingspans, respectively (females, *n* = 12 trials across 5 flies; males, *n* = 29 trials across 7 flies). Neurons are activated using a 2 mW/cm^2^ 617 nm light delivered at the neuron focal plane. Panels show mean ± standard error of mean.

**Figure 8.**
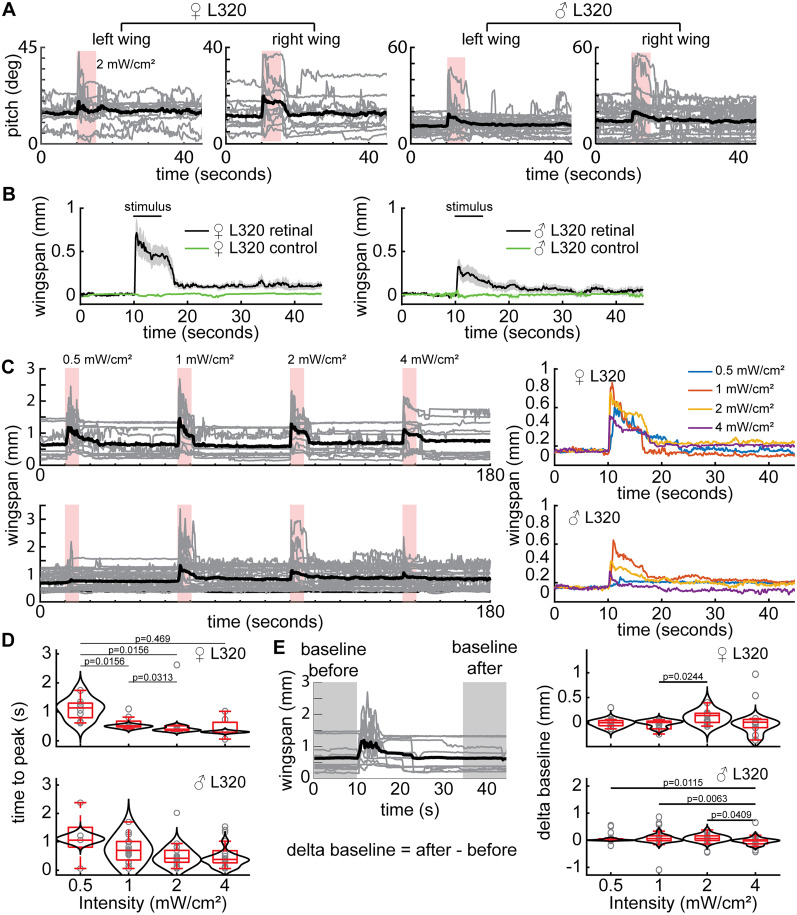
Optogenetic stimulation in head-fixed open cuticle flies is capable of driving wing behavior. ***A***, Single-trial left- and right-wing pitch of L320 > UASChrimson flies in a dissected head-fixed trackball setup. A 2 mW/cm^2^ 617 nm light is delivered during the 5 s stimulus period (females, *n* = 11 trials across 5 flies; males, *n* = 26 trials across 7 flies) ***B***, L320 > UASChrimson flies not fed with retinal do not display behavior (control females, *n* = 20 trials across 5 flies; control males, *n* = 20 trials across 5 flies). Panels show mean ± standard error of mean. ***C***, Same as ***A***, but for wingspan. Each trial comprises of four increasing light intensities with a 30 s rest period in between. Left, All trials across female (top) and male (bottom) flies. Right, Mean wingspan averaged across trials based on stimulus intensity for females (top) and males (bottom). Trials were baseline subtracted and aligned by 10 s before the stimulus onset. ***D***, Latency to the first the peak in wingspan after stimulus light on for female (top) and male (bottom) flies. Trials where the wingspan did not change were not considered (Wilcoxon rank-sum test; females, *n* = 8, 8, 8, 7 trials; males, *n* = 5, 19, 15, 17 trials for 0.5, 1, 2, and 4 mW/cm^2^, respectively). ***E***, Left, Schematic illustrating the change in baseline wingspan in the time period 10 s before the stimulus onset and 20–30 s after the stimulus offset. Right, Change in baseline wingspan for female (top) and male (bottom) flies (Wilcoxon signed-rank test; females, *n* = 11 trials; males, *n* = 26 trials for 0.5, 1, 2, and 4 mW/cm^2^). A complete list of *p* values and *W* values for ***D*** and ***E*** are in Table 2.

To determine which population of neurons is important for driving aggression, we used a DMD projector to optogenetically activate each cluster of neurons independently (Fig. 7*A*). Since in this setup, flies are head-fixed and walk on an air-supported ball and aggressive behaviors have never been demonstrated in a head-fixed preparation, we first characterized the effect of activating all L320 neurons. Using light delivered at the image plane, both male and female flies elicited a robust wing response; as in the case of freely walking flies, the wing response was characterized by a change in wing pitch (Fig. 7*B*). Perhaps due to variability between preparations, the baseline wing pitch was different across flies (Fig. 8*A*; Movie 3) and led to different physical space available for each wing which would account for some of the variability in behavior; despite these differences, flies showed wing expansion in most trials. Because the main purpose of this experiment was to identify which population of neurons caused aggressive wing threat displays, we chose a simple metric (Fig. 7*A*)—wingspan which we defined as the distance between the two wing tips—to analyze the effect of activating a given population of neurons. Activating all L320 neurons causes a large change in wingspan (Fig. 7*B*) which is not observed in the control nonretinal flies (Fig. 8*B*). We also measured the effect of different light intensities and found that female flies appear more sensitive than male flies and will elicit robust wingspan increase at a light intensity (0.5 mW/cm^2^) at which males show only a weak and delayed wing threat (Fig. 8*C*). As the light intensity increases, latency to the initial wing response becomes faster (Fig. 8*D*), and the persistence of the wing response after the stimulus is turned off increases (Fig. 7*E*). A 1 mW/cm^2^ stimulus is sufficient to drive a robust wing response in both male and female flies with low latency and no change in baseline activity. When the stimulation intensity was increased to 2 mW/cm^2^, both male and female flies did not return to their initial completely closed-wing state. Rather, they held their wings slightly ajar for over 30 s (Fig. 8*C*,*E*). Finally, after stimulation with a 4 mW/cm^2^ stimulus, the increase in wingspan becomes less robust and will sometimes not elicit wing movement among female flies (Fig. 8*C*). We utilized the 2 mW/cm^2^ stimulus to study the role of different neural populations because it generated the most robust response (Fig. 8*C*).

**Figure 9.**
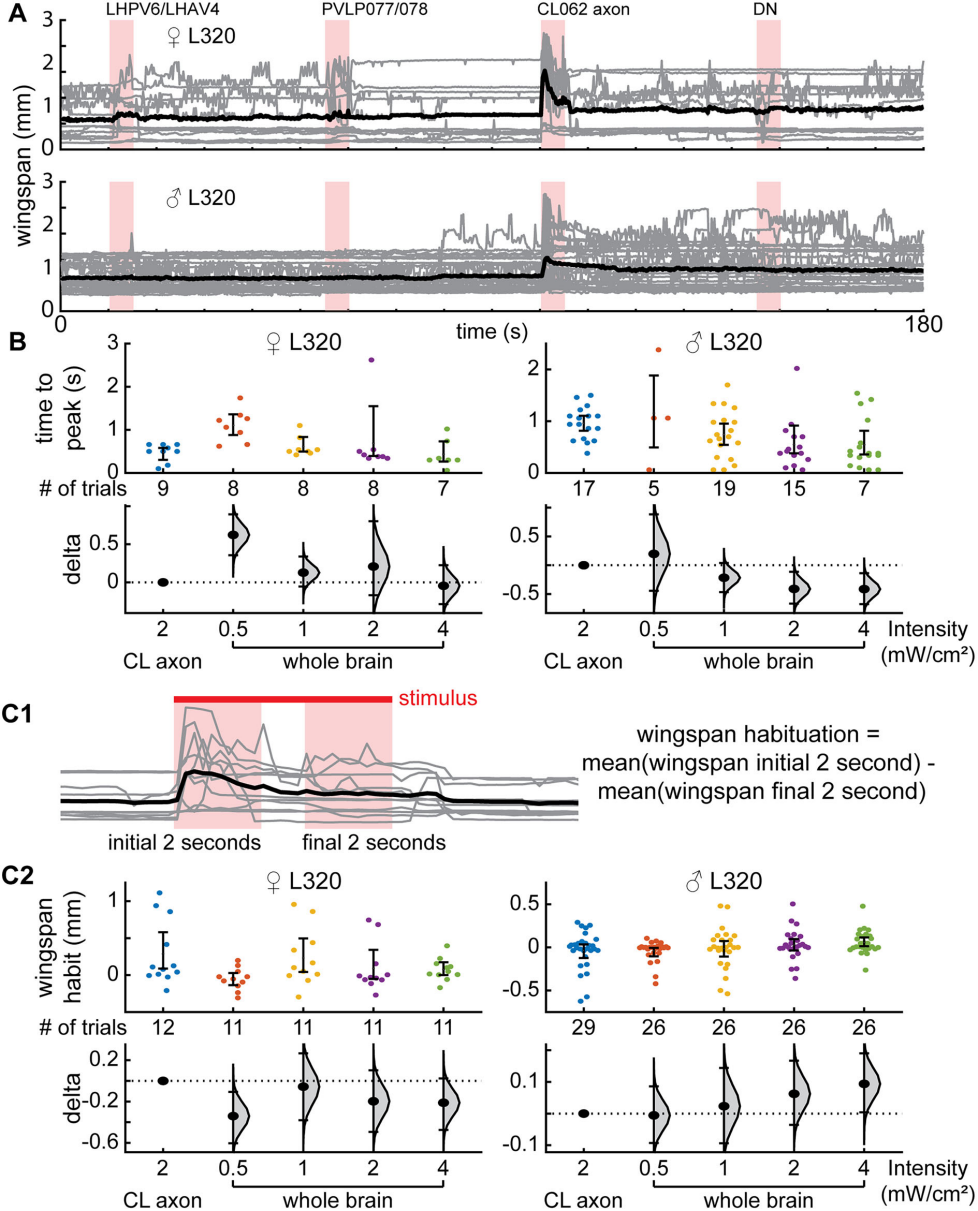
Activation of only CL062 axons drives wing behavior. The level of this behavior is similar to the activation of all L320-split neurons at half the light intensity. ***A***, Wingspan of UASChrimson > L320-split-gal4 flies in a dissected head-fixed trackball setup. Each trial comprises of sequentially activating the LH neurons, the PVLP neurons, the CL062 axons, and finally the DN. Neurons are activated using a 2 mW/cm^2^ 617 nm light through a DMD projector. There is a 30 s rest period between each stimulus application. All trials across female (top) and male (bottom) flies (males, *n* = 29 trials across 7 flies; females, *n* = 12 trials across 5 flies). ***B***, Latencies to the peak of wingspan after stimulus light on for female (left) and male (right) when the CL062 axons were targeted with a 2 mW/cm^2^ 617 nm as compared with optogenetic stimulation of all neurons are different intensities. Trials where the wingspan did not peak were not included. ***C*1**, Definition of wingspan habituation as the difference in mean wingspan during the first 2 s of each stimulus bout minus the mean wingspan during the last 2 s of each stimulus bout. ***C*2**, Wingspan habituations for female (left) and male (right) when the CL062 axons were targeted with a 2 mW/cm^2^ 617 as compared with optogenetic stimulation of all neurons are different intensities.

To assess which L320 neurons drive wing threat, we activated the LH, PVLP, CL062, and DN by changing the area over which the red light is turned on (Fig. 7*C*). Since it was difficult to isolate the cell bodies of CL062 somas from the neurite of the LH and PVLP neurons within our preparation, we targeted the midline crossing portion of the CL062 axons (blue oval). We found that activation of the CL062 axons, but not the other neurons, drove a robust increase in wingspan (Figs. 7*C*, 9*A*). The latency to the initial peak in wingspan and the wingspan habituation when only the CL062 axons are stimulated was most similar to that observed when performing brain-wide stimulation of all L320-split neurons at approximately half the light intensity (Fig. 9*B*,*C*). Since channelrhodopsins, such as CsChrimson, can be expressed throughout the neuron and the midline crossing portion of the CL062 neurons that we targeted represents approximately half of the axon, this weaker behavioral response may be a consequence of activating only a subset of all Chrimson channels. Finally, we did observe a small increase in the wingspan in female flies when activating the LH and PVLP clusters of neurons (Fig. 7*C*). This is likely due to off-target activation of CL062 somas since they are in close spatial proximity to the LH and PVLP neuron neurites within our experimental preparation.

**Figure 10.**
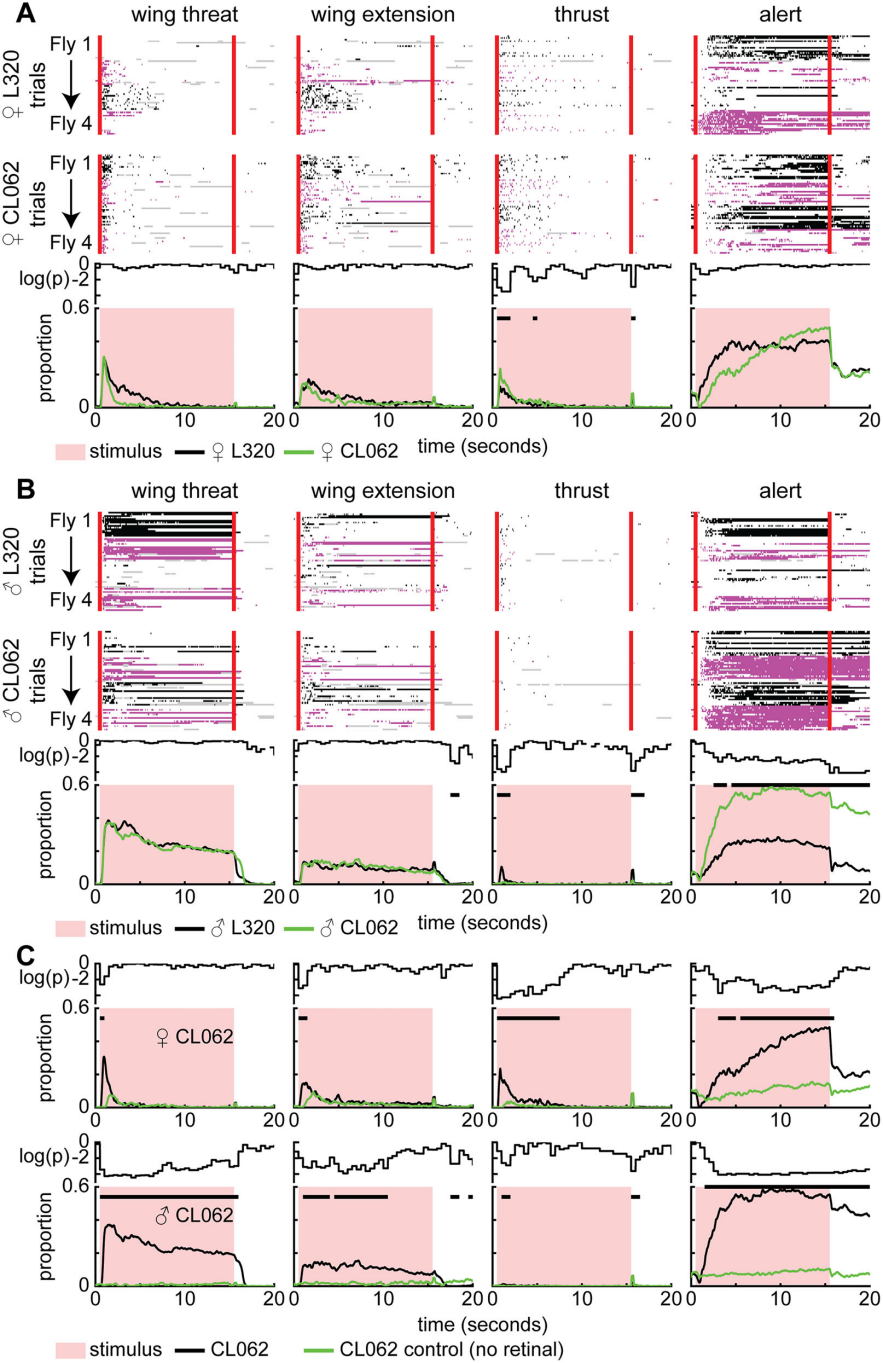
Activation of CL062 neurons using a more restrictive line reproduces L320 behavior. ***A***, Top rows, Single-trial ethogram of actions for four-sample female L320 > UASChrimson and CL062-split > UASChrimson flies. Trials grouped by flies (Fly 1, Trials 1–15; Fly 2, Trials 16–30; etc.). The red line indicates the start and end of optogenetic stimulation period, which lasts for 15 s. Alternating black and purple blocks of trials represent different flies. Gray areas indicate frames where any of the tracked body parts used to identify the action had low tracking confidence (see Materials and Methods). Bottom row, Proportion of trials where flies are performing each action. An 8.9 mW/cm^2^ 617 nm light is turned on during the stimulus period (retinal fed, *n* = 12 L320 flies; *n* = 11 CL062 flies; 15 trials/fly). Black bars show time points where there is a significant difference between L320 > UASChrimson and CL062-split > UASChrimson flies (Wilcoxon rank-sum test *p* < 0.01; see Materials and Methods). ***B***, Same as ***A*** but for male L320 > UASChrimson and CL062-split > UASChrimson flies (retinal fed, *n* = 11 L320 flies; *n* = 11 CL062 flies; 15 trials/fly). ***C***, Top row, Proportion of trials where female CL062-split > UASChrimson flies not fed with retinal are performing each action (retinal fed, *n* = 11 flies; control, *n* = 12 flies; 15 trials/fly). Black bars show time points where there is a significant difference between retinal fed and control flies (Wilcoxon rank-sum test *p* < 0.01; see Materials and Methods). Bottom row, Same as the top row but for male CL062-split > UASChrimson flies (retinal fed, *n* = 11 flies; control, *n* = 11 flies; 15 trials/fly). For ***A–C***, the top row shows log10 of the *p* value.

**Figure 11.**
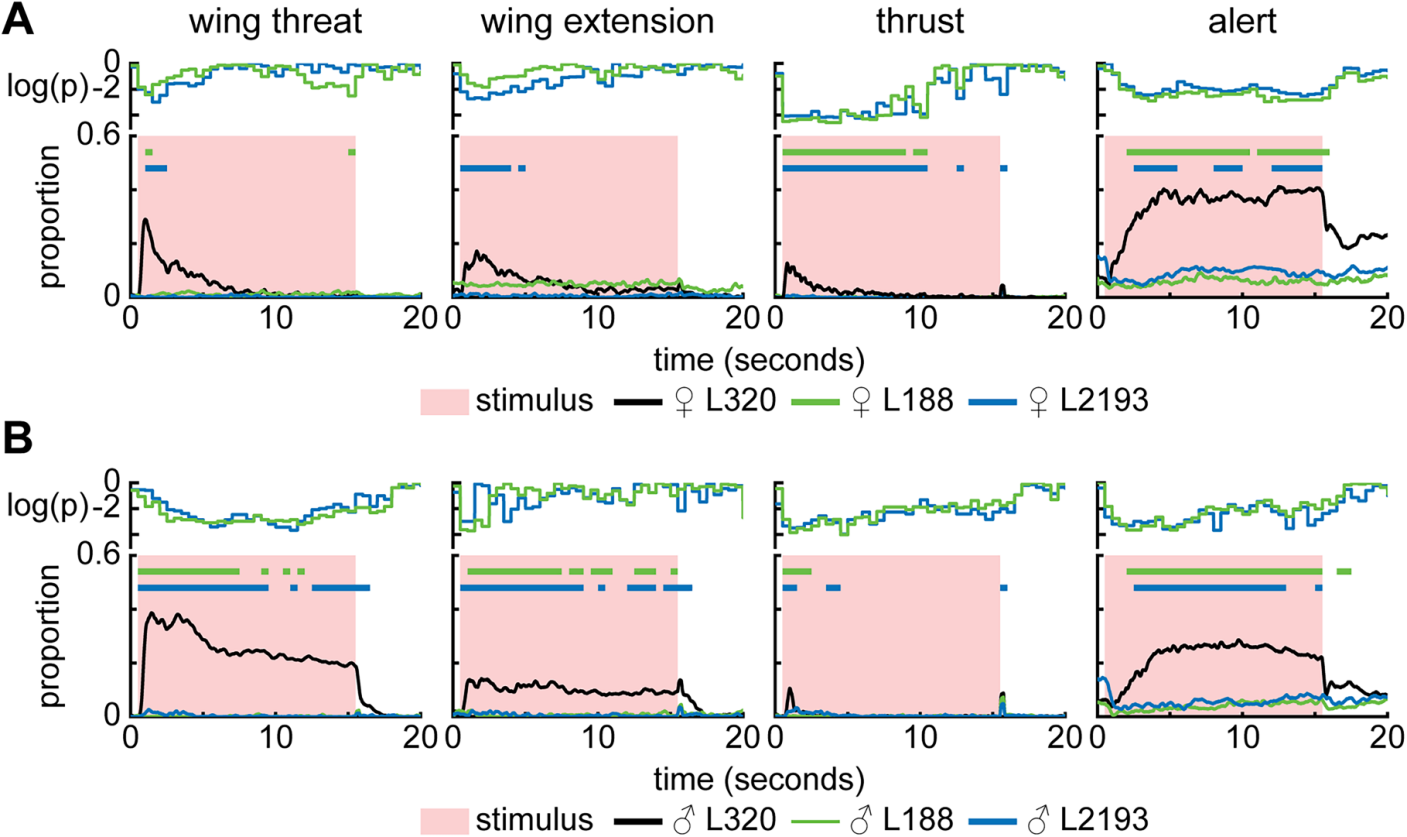
LHPV6a1/3 does not drive aggressive actions. ***A***, L188 split-GAL4 and L2193 split-GAL4 labels LHPV6a1 and LHPV6a3 neurons, respectively. Shown are the proportion of trials where female L188 split-GAL4 > UASChrimson and L2193 split-GAL4 > UASChrimson flies are performing each action. An 8.9 mW/cm^2^ 617 nm light is turned on during the stimulus period (retinal fed, *n* = 11 L188 flies; *n* = 10 L2193 flies; 15 trials/fly). Green and blue bars show time points where there is a significant difference between L320 > UASChrimson and L188 > UASChrimson and L2193 > UASChrimson flies, respectively (Wilcoxon rank-sum test *p* < 0.01; see Materials and Methods). ***B***, Same as ***A*** but for male flies (retinal fed, *n* = 9 L188 flies; *n* = 10 L2193 flies; 15 trials/fly). For ***A*** and ***B***, the top row shows log10 of the *p* value.

To assess whether the behavior elicited by the L320 line in freely walking flies resulted from the activation of the CL062 neurons, we utilized a previously reported split-GAL4 line called Split^Thr^ (GMR22D03-AD ∩ GMR20E08-DBD; Duistermars et al., 2018) that has been shown to drive male wing threats. We will refer to this line as CL062-split since it labels only the CL062 neurons and a small population of dorsal neurons that are not implicated in the behavior. Both female and male CL062-split flies performed the same set of actions; the time-course of the actions is similar to that of L320-split flies (Fig. 10, Movie 4). Despite the similarities, there are some differences in the temporal dynamics of the probability of observing lunges and the alert stance. Female CL062-split flies show a higher propensity to thrust immediately after the stimulus onset, while male CL062-split flies did not perform thrusts (Fig. 10*A*). A higher proportion of male CL062-split flies also stood in the alert stance throughout the stimulus period (Fig. 10*B*). Female flies not fed retinal displayed a muted amount of wing extension and alert stance, perhaps reflecting leaky optogenetic activation of neurons (Fig. 10*C*). To confirm that the small increase in wingspan observed when we performed spatially restricted targeting of LH neurons was due to off-target activation of CL062 neurons and not the LH neurons themselves, we utilized two split-Gal4 lines called L188 (GMR47B03-AD ∩ GMR30H02-DBD) and L2193 (VT060077-AD ∩ VT029317-DBD) that label subsets of the LH neurons expressed by L320-split. L188 labels LHPV6a1 while L2193 labels LHPV6a3 neurons (Dolan et al., 2019). Optogenetic activation of these genetic lines did not result in wing threat, extension, lunging, or holding an alert stance (Fig. 11). It is important to note that activating LH neurons labeled by these lines did result in other behaviors. As these behaviors appear unrelated to aggression, we have not quantified them in this study. Taken together, these experiments suggest that the CL062 neurons are sufficient in driving wing threat, wing extension, thrusting, and alert stance in a temporally structured manner.

### Unilateral activation of CL062 drives bilateral wing threat followed by contralateral wing extension

**Figure 12.**
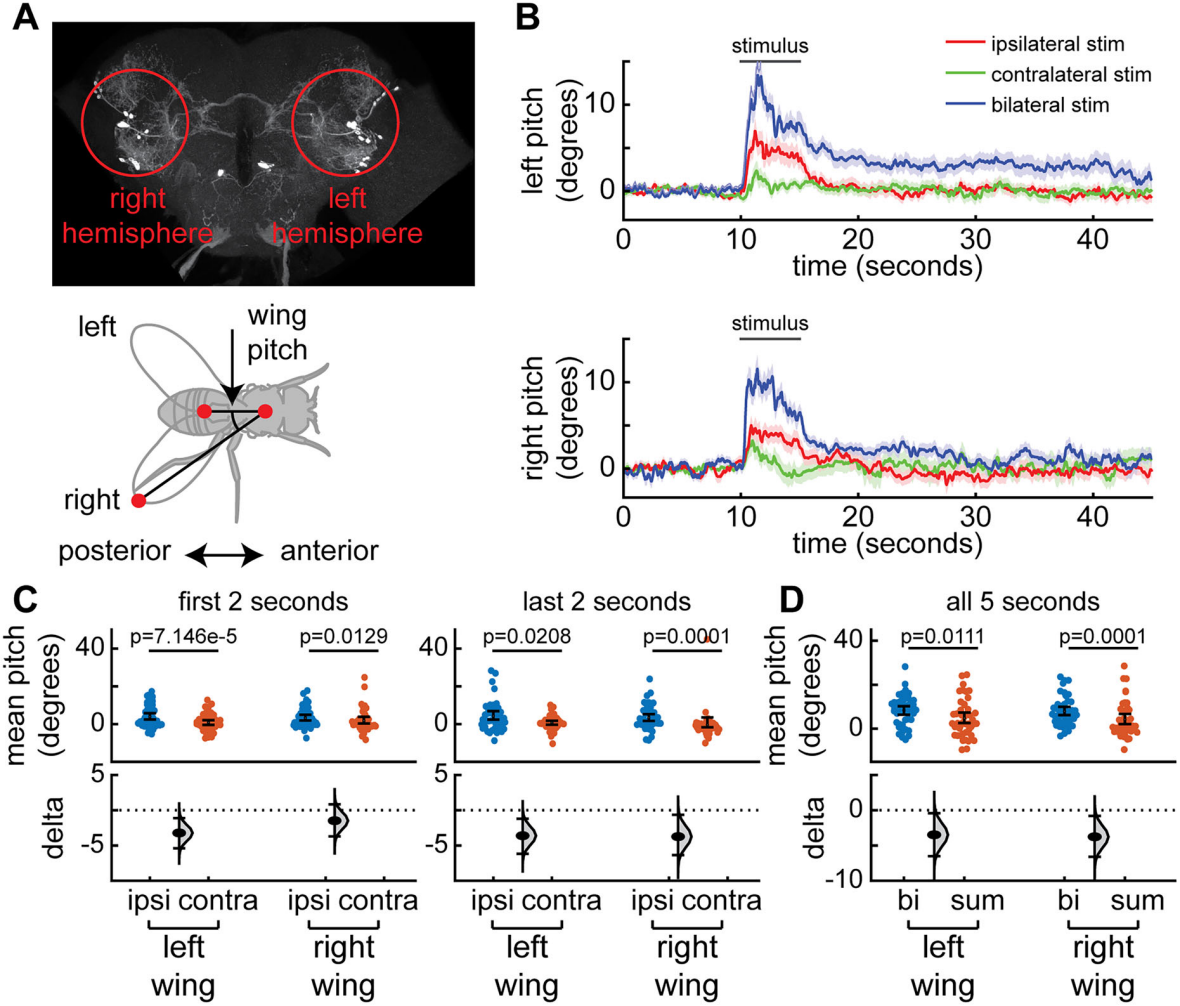
Unilateral activation CL062 neurons shows that ipsilateral and contralateral wings threats are controlled differentially. ***A***, Top, Experimental setup. Red circles show approximately the targeted regions during experimentation. Here, the anterior axis is facing out of the page. Bottom, Top–down diagram of the fly showing the left- and right-wing pitch. ***B***, Baseline-subtracted wing pitch (both left and right) after ipsilateral, contralateral, and bilateral stimulation shows a fast increase in bilateral wing pitch followed by higher ipsilateral wing pitch (females, *n* = 23 trials across 5 L320 > UASChrimson flies). Panels show mean ± standard error of mean. Neurons are activated using a 2 mW/cm^2^ 617 nm light delivered at the neuron focal plane. ***C***, Left, The baseline-subtracted mean left- and right-wing pitch during the first 2 s of ipsilateral stimulus is higher than that of the contralateral stimulus despite bilateral wing pitch changes. Right, The baseline-subtracted mean left- and right-wing pitch during the last 2 s of the ipsilateral stimulus is higher than that of the contralateral stimulus. ***D***, The baseline-subtracted mean pitch during the entire 5 s bilateral stimulation period is significantly higher than the sum of the mean left and right pitch during the stimulus period (Wilcoxon signed-rank test, same trials as in ***B***). A complete list of *p* values and *W* values for ***C*** and ***D*** are in Table 2.

The fact that these neurons can drive both independent movement of the wings and the extension of a single wing for long durations (Fig. 4) is surprising given that CL062 axons project to both hemispheres (Fig. 6). To understand the source of wing extension, we performed unilateral activation of CL062 neurons in female flies (Fig. 12*A*; Movie 5). We found that unilateral stimulation drove an increase in ipsilateral wing pitch (Fig. 12*B*,*C*). Surprisingly, like the ipsilateral wing, the contralateral wing also exhibited an initial increase in wing pitch that quickly habituates within 2 s. This response suggests a multitimescale control of wings initiated by CL062 neurons: Initial activation of these neurons first drives a wing threat response utilizing both wings; the contralateral wing response habituates leaving only the ipsilateral wing extended; this long-lasting ipsilateral wing response is reminiscent of wing extension behavior observed during behavior in freely walking flies. Interestingly, the behavioral response during bilateral activation of CL062 neurons is greater than the sum of unilateral activation, suggesting a potential form of nonlinearity (Fig. 12*D*).

### CL062 likely drives downstream behaviors through multiple parallel DN pathways

**Figure 13.**
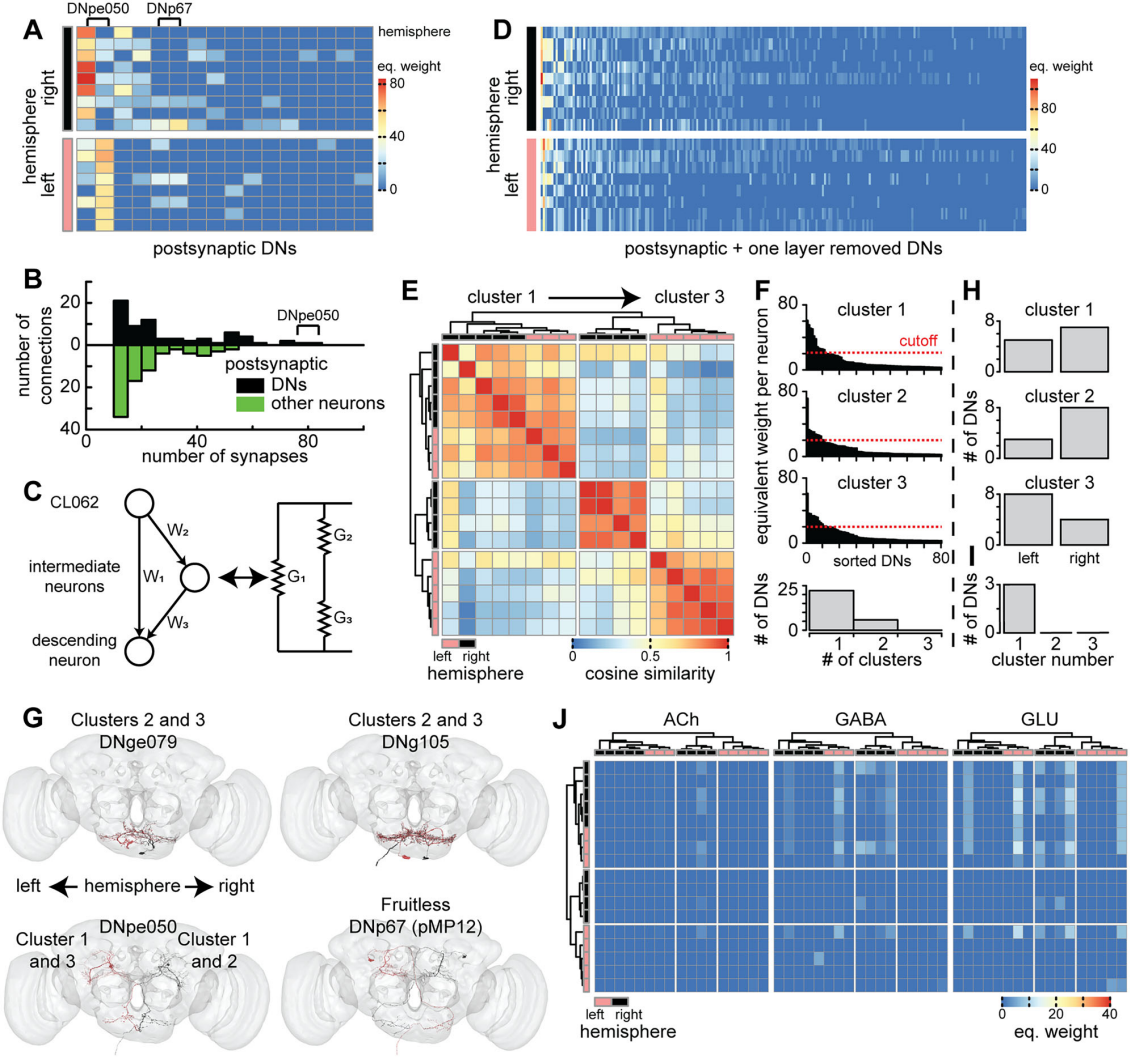
Modularity in CL062 connections to DNs. ***A***, Number of synapses from each CL062 neuron to all direct postsynaptic DNs with at least 10 synapses. ***B***, Number of synapses CL062 neurons make with DNs in comparison with all postsynaptic neurons. ***C***, Schematic of equivalent neuron weights with up to a single intermediate neuron within a path. Single path weight is modeled as conductance of resistors in series. Equivalent weight is modeled as conductance of resistors in parallel. ***D***, Equivalent weight from each CL062 neuron to all connected DNs. ***E***, Hierarchical agglomerative clustering of the cosine similarity between each pair of CL062 neuron to DN connections. There are approximately three clusters of CL062 neurons. ***F***, Sorted equivalent weights per neuron for each of the five clusters shows that each cluster connects strongly to a small number of DNs. A threshold of 20 was used to binarize strongly connected DNs. Bottom, Most DNs are connected strongly to only a single cluster. ***G***, Trends in DN organization based on connections to CL062 neurons. Top row, Two pairs of DNs in the gnathal Ganglia are strongly connected to Cluster 2 and 3 CL062 neurons. Bottom left, DNpe050 is strongly connected to ipsilateral clusters (2 and 3) of CL062 neurons and to the bilateral cluster of CL062 neurons (1). Bottom right, The pair of fruitless DNs predicted to be strongly connected to Cluster 1 CL062 neurons. ***H***, The number of strongly connected DNs within each hemisphere to each cluster of CL062 neurons. ***I***, Number of strongly connected DNs that putatively express fruitless for each cluster. ***J***, Neurotransmitter pathways from CL062 neurons to each other show weak and sparse GABA and glutamate pathways between CL062 neurons. CL062 clusters based on ***E***.

Since activation of CL062 neurons drives multiple actions, we next asked how the CL062 neurons are connected to downstream circuits to drive action. One possibility is that different CL062 neurons drive different subsets of actions in a modular manner. Action choice must be relayed from the brain to motor circuits in the ventral nerve cord through ∼1,100 DNs (Hsu and Bhandawat, 2016). Therefore, if CL062 neurons drive actions in a modular manner, we would expect that different CL062 neurons will have stronger connections to different subsets of DNs. To examine whether there is modularity, we used FlyWire and natverse toolsets to obtain the connectivity of DNs that are postsynaptic to CL062 (Heinrich et al., 2018; Bates et al., 2020; Buhmann et al., 2021; Dorkenwald et al., 2022). These connections are based on a fully reconstructed female FAFB and curated by the FlyWire community (Zheng et al., 2018; Dorkenwald et al., 2022; Schlegel et al., 2023; Sven et al., 2023). We found that each CL062 neuron makes at least 10 synapses with four DNs on average (Fig. 13*A*). Although CL062 makes connections to non-DNs, the strong connections are more likely to be to DNs (Fig. 13*B*) with these DNs making up ∼53.4% of total synaptic connections at this threshold. Only a single pair of DNs, DNpe050, is postsynaptic to every ipsilateral CL062 and most contralateral CL062 neurons (Fig. 13*A*). The pair of DNpe050 neurons receives the largest output from the CL062 neurons when the outputs from all the CL062 neurons are summed together (Fig. 13*B*). Besides this pair of DNs, the other DNs are postsynaptic to only small subsets of CL062 neurons within a single hemisphere; these DNs can be either ipsilateral or contralateral (Fig. 13*A*). Finally, there is a single pair of DNs called DNp67 (one of the pMP12) that putatively expresses *fru* according to the connectome data explorer Codex (Matsliah et al., 2023), a gene important for sexually dimorphic social behaviors (Gill, 1963; Vrontou et al., 2006). In a previous study, CL062 neurons have been shown to be connected to pMN1/DNp13, but these connections are through a smaller number of synapses and are not further considered in this study (Baker et al., 2022).

Despite being strongly connected to DNs, CL062 neurons also connect strongly to multiple interneurons, which may in turn connect to DNs (Fig. 13*B*). These forms of indirect connections have been shown to be important for linking structure to functional correlation across the brain (Turner et al., 2021; Uzel et al., 2022). Therefore, we utilized a method based on resistor circuits to determine equivalent feedforward connection weights to all DNs with 0–1 intermediate neuron in between (Fig. 13*C*; see Materials and Methods). There are two main features of this methodology. First, the equivalent weight is higher for DNs that can be reached via multiple intermediate neurons (i.e., parallel pathways). Second, the weight of a pathway is limited by the lowest edge weight between two neurons in the path. While CL062 can connect to a broader set of DNs through second-order connections, we found that these connections are still sparse and modular (Fig. 13*D*). We quantified the similarity in CL062 neurons’ equivalent connections weights to DNs using cosine similarity and found that there are three clusters of CL062 neurons (Fig. 13*E*).

Since the clustering is based on cosine similarity, the presence of multiple clusters of CL062 neurons can be due to either distribution shape or modularity in DN connection identity. In the former case, a CL062 neuron that connects broadly to a distributed set of many DNs will have low cosine similarity to another CL062 neuron that connects specifically to a few DNs even if these DNs are strongly connected to both CL062 neurons. In the latter case, two CL062 neurons that have the same distribution of connections to different populations of DNs will have a low similarity. We found that CL062 neurons in each cluster make a similar distribution of connections to different DNs. Using an equivalent weight threshold of 20 per CL062 neuron, we found that each cluster is strongly connected to ∼12 DNs (Fig. 13*F*). Of these, most DNs make strong connections to only a single cluster of CL062 neurons, and only three pairs of DNs make strong connections to two CL062 clusters (Fig. 13*F*,*G*). Furthermore, Cluster 1, which is comprised of CL062 neurons from both hemispheres, is strongly connected to DNs in both hemispheres (Fig. 13*H*). Meanwhile, Clusters 2 and 3, which are comprised of single hemisphere CL062 neurons, are connected to more ipsilateral than contralateral neurons. Surprisingly, only Cluster 1 CL062 neurons are strongly connected to the fruitless DNp67 (Fig. 13*G*,*I*).

The connectivity pattern that we observe here is consistent with a modular organization in the connections between CL062 neurons and DNs. We hypothesize that activating subsets of CL062 neurons will likely drive subsets of action. Since modular circuits often show mutual inhibition, we assessed the possibility of mutual inhibition between CL062 neurons as a mechanism for shaping the temporal progression of aggressive actions. Since CL062 neurons are all cholinergic, we again considered two-layered connections between CL062 neurons. We found that CL062 neurons make sparse glutamatergic and GABAergic connections with each other (Fig. 13*J*) consistent with weak mutual inhibition. In flies, GABA is the primary inhibitory neurotransmitter, while glutamate has been shown in some visual and olfactory circuits to be inhibitory (W. W. Liu and Wilson, 2013; Molina-Obando et al., 2019). Thus, the evidence for mutual inhibition is not strong; and the weak mutual inhibition is consistent with the idea that aggressive actions elicited by CL062 do not reflect strict progression and that multiple actions such as (wing threat and thrusts) can occur at the same time.

### CL062 and aIPg connect to different sets of DNs and CL062 sparsely inhibits aIPg through the inhibition of pC1d

How are the CL062 neurons related to known aggression neurons? To date, most neurons that mediate aggression are known to be part of the *fru*^+^/*dsx*^+^ circuit. In females, two interconnected populations of *fru*^+^ neurons pC1d and aIPg are important for aggression (Schretter et al., 2020). Are the CL062 neurons also part of the same circuit and either upstream or downstream to those neurons? It is also possible that aIPg/pC1 neurons are mutually antagonistic to the CL062 neurons: During aggression, after approaching, female flies will transition into either a wing threat or a headbutt. The choice of this action initiates two distinct action sequence loops (Nilsen et al., 2004). One action sequence loop involves wing threat and thrusting, while the other involves headbutting and multiple types of fencing actions. It is possible that a mechanism for these two loops is mutual antagonism between CL062 which drives wing threat and aIPg that drives headbutting and fencing actions (Schretter et al., 2020).

**Figure 14.**
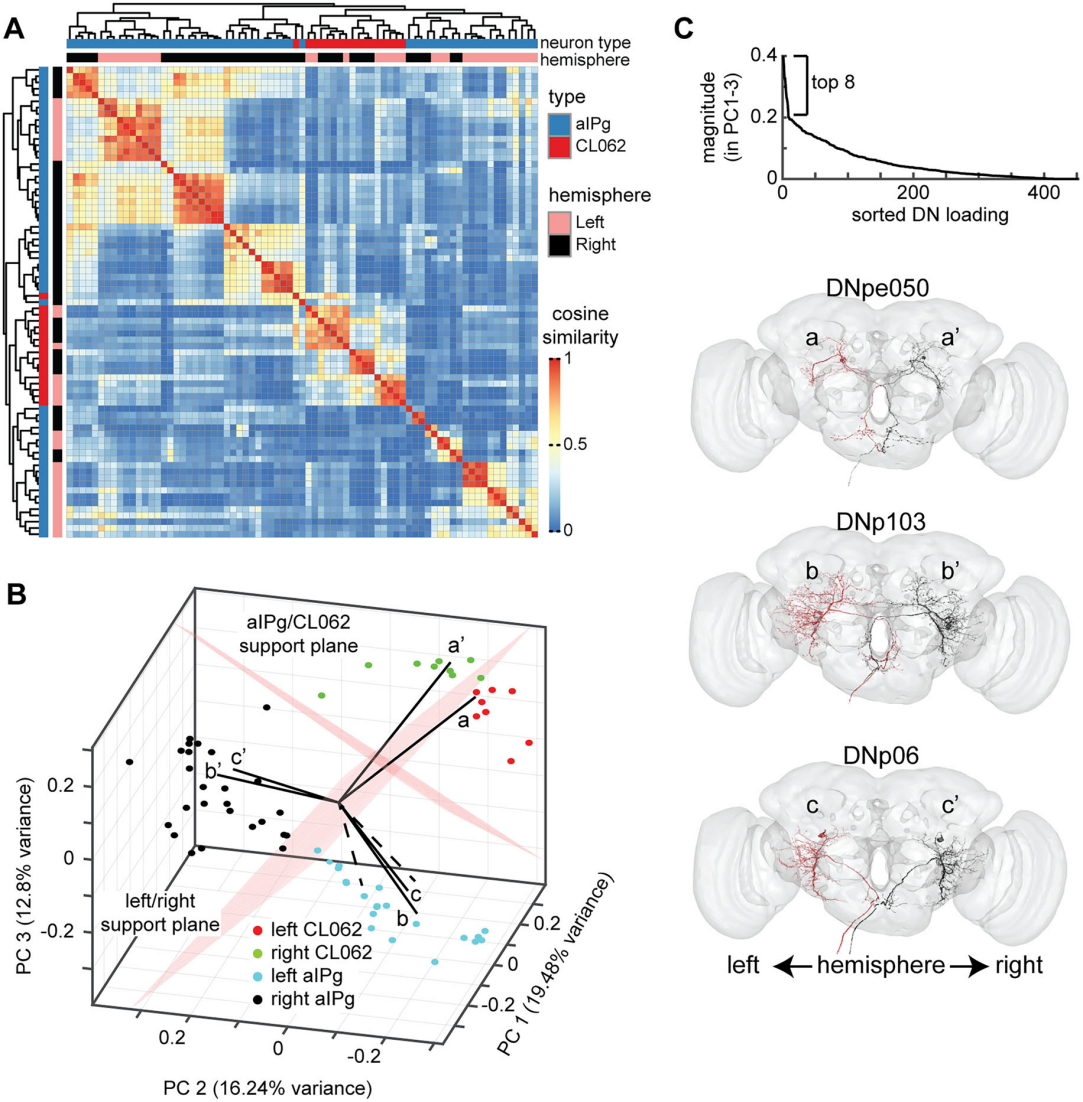
CL062 and aIPg likely drive actions through parallel DN pathways. ***A***, Hierarchical agglomerative clustering of the cosine similarity between each pair of CL062 and aIPg neurons to DN connections. CL062 does not make connections to similar DNs as aIPg. ***B***, Biplot of the first three PCs of L2 normalized connections to DNs from CL and aIPg neurons. The first three PCs appear to separate left from right hemisphere neurons as well as aIPg from CL. The two support planes are nearly orthogonal (87.9°). ***C***, Top, Magnitude of loading vectors in the 3D PC space show a sharp drop off after the first eight. Of these eight DN loadings, there are three pairs of DNs in the direction of CL062, left aIPg, and right aIPg neuron clusters (***B***, solid lines). Bottom, Based on the direction of their loadings, these neurons are most strongly connected with ipsilateral CL and aIPg neurons, respectively.

In either case, it is likely that they also connect to different sets of DNs. Like CL062, aIPg neurons make connections to multiple DNs through one layer of intermediate neurons. If CL062 and aIPg are involved in circuits driving different actions during aggression, then we should find that they connect to different subsets of DNs. Indeed, when we compared the cosine similarity between all CL062 and aIPg neurons, we found that there is a low similarity between aIPg and CL062 neurons (Fig. 14*A*). We next sought to determine the set of DNs that may be important for the separation of the action sequence loops by determining the set of DNs that contribute highly to segregating aIPg neurons from CL062 neurons. To accomplish this, we performed PCA to find principal components (PCs) that explain most of the variance in the connections from aIPg and CL062 neurons to DNs. We found that the first three PCs partitioned the left and right hemisphere aIPg and CL062 into different clusters (Fig. 14*B*). We can construct a pair of near orthogonal support planes in this space that defines a left/right axis and an aIPg/CL062 axis. The largest contributions of DNs in the direction of left and right hemisphere CL clusters are close to the left/right support plane (Fig. 14*B*,*C*) reflecting that these DNpe050 neurons are the only pair of DNs that connect strongly to all ipsilateral and most contralateral CL062 neurons. Meanwhile, the largest loadings of DNs in the direction of the left and right hemisphere aIPg axis lie further away from the left/right support plane implying that these DNs are likely strongly connected with single hemisphere aIPg neurons.

**Figure 15.**
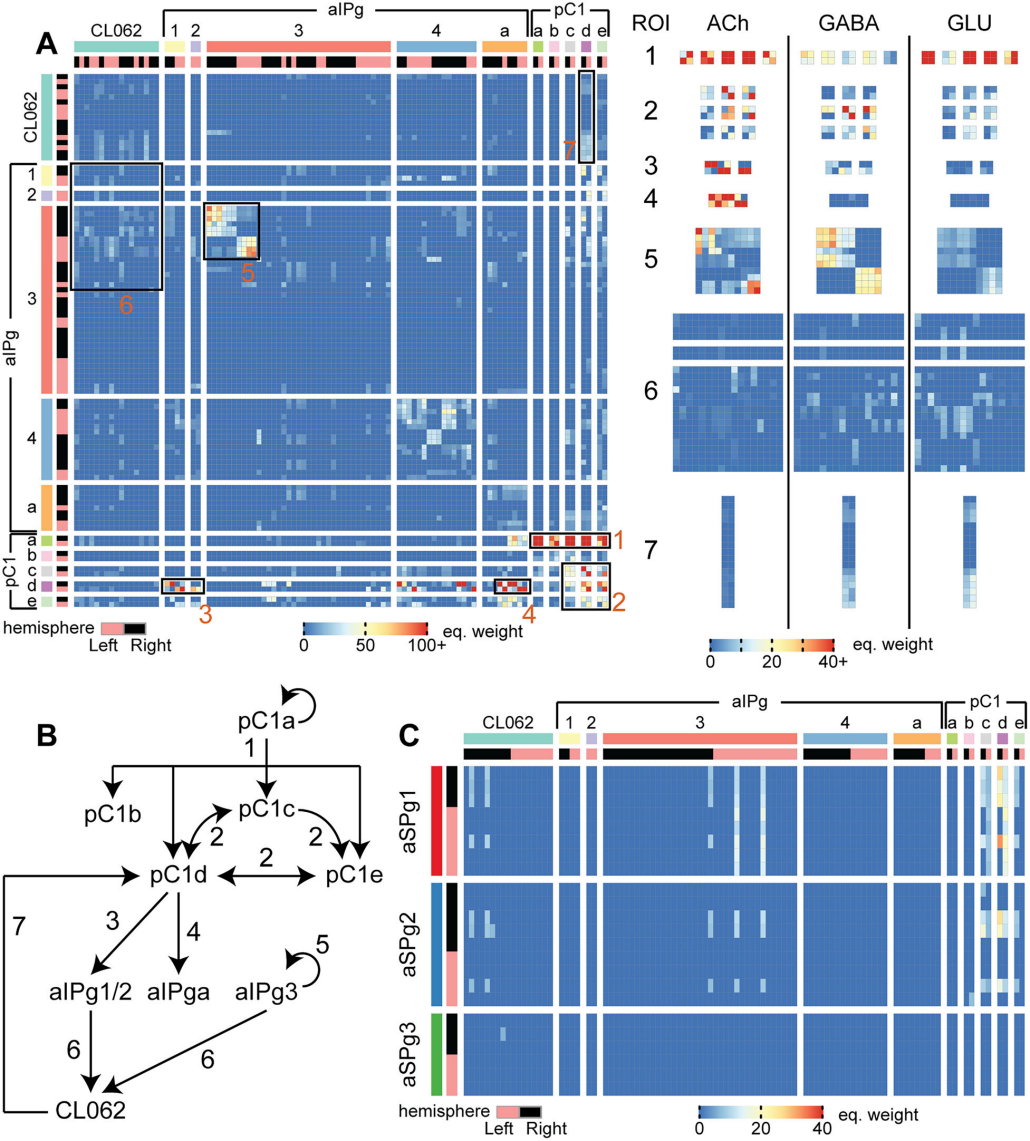
Potential pathways between CL062, aIPg, and pC1 neuron classes. ***A***, Left, Weighted adjacency matrix (row→column) showing equivalent weight between CL062, aIPg, and pC1 neurons. Seven regions are highlighted. Right, The equivalent weights for each of the seven regions broken down by neurotransmitter pathway. Since CL062, aIPg, and pC1 neurons are all cholinergic, GABA and glutamate pathways arise from indirect pathways. ***B***, Schematic showing the subset of potential pathways between neuron classes highlighted by the seven ROIs (numbers). Arrows indicate directionality. ***C***, aSPg makes connections to pC1 and sparse connections to CL062. Matrix (row→column) showing equivalent weight from aSPg to CL062, aIPg, and pC1 neurons. Pathways in panels ***A–C*** include both direct connections and one layer removed indirect connections via an intermediate neuron.

Since CL062 and aIPg neurons drive different actions and connect strongly to different subsets of DNs, we next considered whether CL062 neurons act to inhibit neural circuits involving aIPg to drive the initial choice of wing threat over headbutting. A past study has found that aIPg receives strong inputs from a class of neurons called pC1d, which also drives a similar phenotype upon optogenetic activation (Palavicino-Maggio et al., 2019; Deutsch et al., 2020; Schretter et al., 2020). These pC1d neurons are part of and are recurrently connected to a group of five pC1 neurons per hemisphere that are involved in multiple social behaviors (L. Wang and Anderson, 2010; Zhou et al., 2014; Koganezawa et al., 2016; Chiu et al., 2021). Since CL062, aIPg, and pC1 neurons are all cholinergic, we again considered two-layered connections between these neuron types. We found that connections between individual CL062 neurons and aIPg/pC1 neurons were sparse (Fig. 15*A*). CL062 neurons form a combination of GABAergic and glutaminergic pathways to pC1d. pC1d in turn excites three types of aIPg (aIPg1, aIPg2, and aIPga) neurons as well as a subset of aIPg3 and aIPg4 neurons. This connectivity pattern suggests a circuit motif where CL062 neurons could inhibit aIPg neurons indirectly through potential inhibition of pC1d. Meanwhile, aIPg neurons are sparsely connected to CL062 through predominantly GABAergic and glutaminergic synapses. Interestingly, a subset of aIPg3 neurons appears to make strong recurrent connections to each other that do not require other aIPg, pC1, or CL062 neurons. Finally, CL062 neurons do not appear to interact strongly with other classes of pC1 neurons even though pC1d appears to take strong feedforward input from pC1a and make strong recurrent pathways with pC1c and pC1e (Fig. 15). In sum, although there are some potential inhibitory interactions between CL062 neurons and the pC1/aIPg circuit, they largely appear to be independent.

**Movie 1.** Example of a female L320 > UASChrimson fly following the optogenetic stimulus onset. The original 100 Hz behavioral video is played back at 5 Hz. Instances where the fly is performing wing threat, wing extension, and thrusting are indicated in the top-left corner. [View online]

**Movie 2.** Example of a male L320 > UASChrimson fly following the optogenetic stimulus onset. The original 100 Hz behavioral video is played back at 5 Hz. Instances where the fly is performing wing threat and thrusting are indicated in the top-left corner. [View online]

**Movie 3.** Example of a female L320 > UASChrimson fly on a floating ball where an optogenetic light is delivered through an epifluorescence microscope directly onto the brain. Left side, Top-left image shows the maximum projection image of the fly brain with the circle representing the region where a 617 nm stimulus light is delivered. The transparency of the circular region represents the intensity, and the actual intensity value is displayed in the top-left corner. The other three left panels show different camera views of the fly with DLC tracking. Open circles are points where there is low confidence in the tracking. Right side, The top row shows two views of the 3D triangulated fly head, thorax, abdomen points, and wingtips. Middle and bottom rows show the wing pitch and wingspan of the fly. [View online]

**Movie 4.** Example of a female CL-Split > UASChrimson fly following the optogenetic stimulus onset. The original 100 Hz behavioral video is played back at 5 Hz. Instances where the fly is performing wing threat, wing extension, thrusting, and standing in an alert posture are indicated in the top-left corner. [View online]

**Movie 5.** Example of ipsilateral versus contralateral stimulation of CL062 neurons in a female L320 > UASChrimson fly on a floating ball. Left side, Top-left image shows the maximum projection image of the fly brain with the circle representing the region where a 617 nm stimulus light is delivered. The transparency of the circular region represents the intensity, and the actual intensity value is displayed in the top-left corner. The other three left panels show different camera views of the fly with DLC tracking. Open circles are points where there is low confidence in the tracking. Right side, The top row shows two views of the 3D triangulated fly head, thorax, abdomen points, and wingtips. Middle and bottom rows show the wing pitch and wingspan of the fly. [View online]

## Discussion

The behavioral experiments and connectomics analysis presented here result in three salient results. First, the CL062 neurons which were previously discovered to elicit aggressive actions in males elicit aggressive actions in both males and females (Duistermars et al., 2018). Although activation of these neurons elicits the same action in both males and females, there are important differences between the male and female behaviors that mirror their aggressive actions in the presence of a target. Second, a target is not necessary for these actions. Third, the CL062 neurons do not express *fruitless* like the preponderance of other aggressive neurons (Duistermars et al., 2018). This lack of expression of genes that mediate much of the sexual dimorphism and the fact that these neurons are also not strongly connected to other known neurons that produce aggression implies that mechanisms underlying aggressive behavior in flies are more distributed than previously thought.

### Activating CL062 neurons in isolated freely walking and head-fixed flies resemble aggressive actions observed in the presence of a mate

Are the actions that we observe here aggressive actions? Both the individual actions observed and their time-course strongly suggest that the behavior observed here is aggression. At the level of individual aggressive actions, wing threats observed here, characterized by elevation of both wings >45°, are a hallmark of aggressive action (Nilsen et al., 2004) and distinct from wing extensions during courtship during which the wings extend horizontally. Strikingly, although both males and females display wing threats upon activation of these neurons, the time-course of the wing threats is sexually dimorphic: In males, wing threats last longer with some wing threat episodes lasting >1 s (Fig. 4). The same long-lasting wing threats have been observed by others (Nilsen et al., 2004; Duistermars et al., 2018). In contrast, female wing-threat bouts rarely lasted longer than 1 s. We observed this dimorphism not only in freely walking flies but also in head-fixed flies.

We also observe the many forms of thrusts reported by others: In some cases, thrusts simply involve a forward movement of the body without any concomitant movement of the legs, at other times; in another form of thrust, the forelegs are lifted, and the body elevates before snapping down; in yet another form, even the middle and back legs are lifted off the ground. These forms of thrusts are similar to those observed by others (Nilsen et al., 2004).

Apart from individual actions, the sequence of actions observed here has a strong resemblance to the sequence observed during agonistic interactions between pairs of flies. Unlike the relative orderly progression during courtship, aggression involves a more complex structure of recurring behavioral sequences (Chen et al., 2002; Nilsen et al., 2004; Hoopfer, 2016). Within a single activation, a given action such as wing threat can occur by itself or along with multiple forms of thrust. Although a given action can occur throughout a trial, the probability of observing a given action changes over time; the change in probability is also sexually dimorphic: Males are more likely to show wing threat throughout the trial, while females are more likely to show threats at the beginning of the trial. In contrast, females are more likely to thrust throughout the trial. These dimorphisms in behavior are reflective of dimorphism observed during natural agonistic interactions.

Taken together, these data suggest that CL062 neurons can not only mediate aggressive actions with a short latency but also accomplish this even in the absence of a target. Given that the individual actions resemble actions during natural aggressive behaviors and the sequence of actions resembles the sequence during natural behaviors, CL062 is likely a monomorphic node that orchestrates aggressive behaviors. The actions and their sequence appear to follow from the activation of this node.

### Implications for the organization of circuits that control aggressive behaviors in *Drosophila*

As aggressive behaviors are social behaviors, sexually dimorphic circuits play an important role in their control. Many of the neurons involved in fly aggression are *fru*^+^/*dsx*^+^ and are important for mediating the choice between other social behaviors such as courtship. Since many social behaviors are dimorphic, these circuits are dimorphic as well. In this light, it is noteworthy that CL062 neurons are neither *fru*^+^ nor are they sexually dimorphic which makes understanding how the CL062 circuit relates to other aggression circuits critical. Since CL062 neurons drive aggressive behaviors in both males and females, there are implications for the circuit organization of aggressive behaviors in both.

Regarding the circuit organization of aggressive behaviors in the females, CL062 neurons appear to function independently of the previously characterized neurons for female aggression, aIPg and pC1d, and the relationship of CL062 to aggression has important differences from these neurons. One important difference is behavioral persistence. Activation of pC1d/e elicits persistent behavior in females (Deutsch et al., 2020; Chiu et al., 2023), in part, through its strong connections to aIPg (Schretter et al., 2020). pC1d/e neurons also drive persistent activity in neurons expressing *Dsx* and *Fru* (Chiu et al., 2023), and their activation produces minutes-long changes in the behavioral state. In contrast, CL062 does not appear to have much long-lasting effect on aggression (Duistermars et al., 2018).

Another important difference is that activation of aIPg and pC1d/e neurons in isolated flies has not been shown to drive aggressive behaviors (Palavicino-Maggio et al., 2019); this lack of acute behavior is another fundamental difference from the CL062 neurons.

One final difference is in the behaviors elicited by the two sets of neurons. In one study, the pC1d and/or aIPg neurons appear to elicit headbutting but not wing threat (Schretter et al., 2020) suggesting that CL062 neurons might be the only ones mediating wing threat. These studies suggest that CL062 and pC1d/aIPg might mediate the two parallel action sequence—one involving wing threat and the other involving headbutting (Nilsen et al., 2004). The presence of potentially inhibitory connections from CL062 to pC1d and from aIPg to CL062 provides a mechanism to inhibit the headbutt sequence loop once the decision for wing threat has been made (Fig. 15). However, in another study, activation of pC1 neurons resulted in both unilateral and bilateral wing extensions (Deutsch et al., 2020). Similarly, thermogenetic activation of R26E01 (broad line) and more restricted subpopulations using an intersectional strategy shows that female flies will perform high posture fencing, headbutting, charging, and wing threats. None of these lines label the CL062 neurons or any fru+ neurons (Palavicino-Maggio et al., 2019) implying that neurons other than CL062 can elicit wing threats. Overall, the data are more consistent with the idea that CL062 represents an aggression circuit independent of the dsx/fru neurons. The fact that connections between CL062 and pC1d/aIPg are sparse supports this idea. The neurons labeled by R26E01 might represent another independent circuit. Although it is formally possible that R26E01 neurons are upstream or downstream of CL062 neurons, this is unlikely because R26E01 only elicits wing threat in females. We hypothesize that there are many such circuits within the fly brain.

CL062—referred to as AIP (anterior inferior protocerebrum) neurons in the other study—has already been implicated in male aggressive behaviors (Duistermars et al., 2018). In the other study, the authors did not observe lunging and concluded that these neurons mediate noncontact aggressive behaviors such as threats and not contact behaviors such as lunges. This discrepancy could arise from several sources. First, there are differences in stimulation protocol between the two studies. In the previous study, the effector was Syn21-Chrimson in vk5 while ours is CsChrimson in attp18. The difference in Chrimson channel, presence of the syn21 translational enhancer, and the location of the landing site can all contribute to the sensitivity discrepancies between the two studies. It is possible that lunges occur at higher activation levels that were not attained in the previous study. Second, we found that activation of just the CL062 neurons exactly replicated the wing threat and wing extension (Fig. 10) behaviors elicited by the much larger L320 neurons quantitatively. However, the thrusts are much lower in males (Fig. 10). Instead, there is a large increase in alert behavior. This difference could be either because of other populations that are labeled in the L320 neurons or because of the additional neurons labeled by L320 in the CL062 cluster. Eight neurons are labeled in L320 versus only 5–6 in CL062-split. Third, it is possible that CL062 neurons are only important for lunging in the context of direct competition for a female during courtship. Consistent with this idea, CL062 neurons are responsive to the pulse component of the male courtship song (Baker et al., 2022). Pulse song can cause male wing threats during competitive pursuit of a female (Y. Jung et al., 2020; Sten et al., 2023). Resolving the issue of whether CL062 neurons contribute to threat displays and lunges or just threat displays will require future loss-of-function experiments in different context in a setup in which wings and other appendages can be tracked in all three dimensions.

Studies published following the discovery of CL062 neurons do not relate these neurons to other aggression circuits (Duistermars et al., 2018; Palavicino-Maggio et al., 2019; Deutsch et al., 2020; Schretter et al., 2020; Chiu et al., 2021). This silence makes it difficult to assess the view of these neurons within the field. It does appear that CL062 is considered unimportant for actual aggression and only important for aggressive displays. Based on inactivation experiments, it has been suggested that CL062 neurons mediate most wing threats (Duistermars et al., 2018). However, this result is inconsistent with other works that have shown that circuits unconnected to the CL062 neurons also seem to mediate wing threats (Certel et al., 2007; Asahina et al., 2014; Duistermars et al., 2018), although this conclusion is based on a single sentence and our interpretation of their result. Essentially, in many studies, wing threat is not characterized. As in the case of females, CL062 neurons likely function in parallel to the well-studied aggression circuits in male—the sexually dimorphic neurons P1a neurons (Bath et al., 2014; Inagaki et al., 2014; Hoopfer et al., 2015; Clemens et al., 2018). The P1a neurons have been shown to drive immediate wing extension through direct activation of a pair of male-specific DNs called pIP10 (von Philipsborn et al., 2011; Clemens et al., 2018; Y. Jung et al., 2020) while activating a recurrent pathway that drives persistent lunging or courtship behaviors (depending on whether a male is present) for tens of minutes after the P1a neurons have stopped being active. The P1a neurons are thought to be the switch in promoting an internal state of aggression or courtship due to this persistence of behavior. Given that P1 neurons are *fru*^+^ and some of their aggressive actions require male-specific DNs, it is likely that they represent a circuit parallel to CL062 neurons. Another set of aggression-producing neurons are the tachykinin-expressing aSPg neurons that promote wing threat, lunging, and tussling toward other males (Asahina et al., 2014). Based on connectomic analysis, these neurons only make sparse connections to the CL062 neurons and are therefore also likely to function in parallel (Fig. 15*C*). In contrast, the tachykinin neurons work together with the P1a neuron to mediate aggression (Hoopfer et al., 2015).

### Hierarchical organization and parallel pathways of aggression

Work in neuroethology postulated hierarchically organized neural circuits underlying sexual behaviors (Tinbergen, 1951; Dawkins, 1976). At the top are “nervous centers” governing reproductive drive. These nervous centers then activate nervous centers governing the choice of competing behaviors such as aggression or courtship. As we descend the hierarchy, nervous centers represent increasingly specific patterns of movement (i.e., actions at Level 3, then movement patterns within an action, then effectors, etc.). Research in the last decade has provided evidence for circuits that underlie this hierarchical organization in multiple model systems. In mice, as postulated by neuroethologists, ventromedial hypothalamus contains dimorphic circuits that are implicated in both aggression and other sexual behaviors (Pfaff and Sakuma, 1979; Lin et al., 2011; C. F. Yang et al., 2013; Lee et al., 2014; Hashikawa et al., 2018; Deutsch et al., 2020). In flies, too, the P1/pC1 cluster is sexually dimorphic and plays a large role in aggression, courtship, mate choice, and egg-laying (L. Wang and Anderson, 2010; Zhou et al., 2014; Hoopfer et al., 2015; Koganezawa et al., 2016; Rezával et al., 2016; Asahina, 2018; Palavicino-Maggio et al., 2019; W. Yang et al., 2019; Ishii et al., 2020; Wohl et al., 2020; Chiu et al., 2021, 2023; K. Wang et al., 2021). Recent work in flies has also shown that activity in these neurons can cause persistent changes in behavior (Hoopfer et al., 2015; Y. Jung et al., 2020; Chiu et al., 2023). Similar persistence has been observed in neurons in the ventromedial hypothalamus that drive multiple defensive behaviors (Kunwar et al., 2015; L. Wang et al., 2015).

Where do the CL062 neurons fall within this hierarchy? Previously, it has been proposed that these CL062 neurons belong to a “Level 3” nervous center since it was found that these neurons promoted noncontact behaviors—such as wing threats, charging, and turning—but not contact actions such as lunging in males (Duistermars et al., 2018). Connectomic results in this study, however, suggest that CL062 are likely to be independent: The set of DNs most likely to be activated by CL062 neurons appear to be different from those activated by the P1/pC1 cluster. A parallel circuit organization is observed even when the circuit is traced through an intervening layer. Moreover, direct connections between P1/pC1 population and CL062 neurons are sparse suggesting that these circuits do not have strong interactions. It is still possible that CL062 are a part of the same hierarchy as the P1/pC1 population, but they are not directly connected or that the small number of direct connections has a disproportional effect.

Another possibility is that CL062 neurons mediate aggression that is not necessarily about competition over resources. As an example, females can occasionally move quickly toward a male with their wings extended as a nonreceptive response to male courtship (Sturtevant, 1915). Similarly, females have been shown to flick their wings and twist their abdomen to escape courting males (Manning, 1960). Thus, it is possible that CL062 neurons mediate other forms of aggression that require a rapid aggressive response rather than a long-lasting change in the state of the fly that characterizes aggression mediated by P1/pC1 neurons. In males, these neurons might play a role in competition during courtship. Consistent with this idea, CL062 neurons have recently been shown to be responsive to male courtship songs (Baker et al., 2022). The existence of an independent circuit for aggression makes sense, as aggression is not a “pure” dimorphic behavior in which males and females exhibit nonoverlapping motor patterns to achieve a similar goal. Aggression is a “mixed” monomorphic–dimorphic behavior in which certain actions are shared and others are dimorphic (Chiu et al., 2021). Recent studies have found a common circuit that mediates approach during aggression and recruits dimorphic circuits during the actual interaction phase of aggression (Chiu et al., 2021). However, many aggressive interactions are similar in males and females and might be mediated by independent circuits such as the CL062 neurons. As noted above, at least one more set of non-CL062, non-P1/pC1 neurons are involved in female fly aggression (Palavicino-Maggio et al., 2019). If CL062 neurons represent an independent aggression-promoting circuit, it is interesting to note that the downstream behavior still exhibits many characteristics of natural aggressive behaviors including sexual dimorphism. Regardless of whether CL062 is independent or not, it is noteworthy to find sexual dimorphism in a fly circuit that might not have a strong interaction with *fru*^+^ neurons.

### Future studies should focus on detailed behavioral characterization

To further assess the organization of aggressive behaviors in flies, a more quantitative assessment is needed. The traditional experimental setup involves a single top–down view which allows for high-throughput screening of social behaviors but sacrifices the ability to observe postural changes such as the body elevation and wing elevation angle (Dierick, 2007; Dankert et al., 2009; Chowdhury et al., 2021). The capacity to observe these postures in behavioral assays with multiple camera views is important for proper classification of aggressive actions. Multiple camera views are particularly important for quantification of wing behavior—particularly for behaviors involving single wing. In a top—down view, it would be difficult to assess whether the extension of a single wing signals courtship or aggression as aggressive wing extensions are indicated by wing elevation. Two camera views are also likely necessary to observe other aspects of behavior such as whether the body angle changed and whether legs were lifted. A thorough characterization of behavior is necessary to understand whether wing threats are completely independent from lunging as is suggested by some (Duistermars et al., 2018). While the wings are often extended during the act of lunging, both actions can occur independently. Second, a precise quantification of body kinematics and postural changes using multiple camera views will be necessary to go beyond the study of the role of neurons/circuits in time-averaged changes to behavior and toward understanding their role in movement-by-movement action selection along with movement. Finally, behavioral characterization in multiple scenarios is necessary to understand aggressive behaviors. Only by activating and inactivating neural circuits in this wider behavioral context can one understand the organization of aggressive circuits.
